# Potassium and sodium microdomains in thin astroglial processes: A computational model study

**DOI:** 10.1371/journal.pcbi.1006151

**Published:** 2018-05-18

**Authors:** Kevin Breslin, John Joseph Wade, KongFatt Wong-Lin, Jim Harkin, Bronac Flanagan, Harm Van Zalinge, Steve Hall, Matthew Walker, Alexei Verkhratsky, Liam McDaid

**Affiliations:** 1 Computational Neuroscience and Neural Engineering (CNET) Research Team, Intelligent Systems Research Centre, Ulster University, Derry, United Kingdom; 2 Neural Systems and Neurotechnology Research Team, Intelligent Systems Research Centre, Ulster University, Derry, United Kingdom; 3 Department of Electrical Engineering and Electronics, University of Liverpool, Liverpool, United Kingdom; 4 Clinical & Experimental Epilepsy Institute of Neurology, University College London, London, United Kingdom; 5 Faculty of Biology, Medicine and Health, University of Manchester, Manchester, United Kingdom; 6 Achucarro Center for Neuroscience, IKERBASQUE, Basque Foundation for Science, Bilbao, Spain; University of Geneva, SWITZERLAND

## Abstract

A biophysical model that captures molecular homeostatic control of ions at the perisynaptic cradle (PsC) is of fundamental importance for understanding the interplay between astroglial and neuronal compartments. In this paper, we develop a multi-compartmental mathematical model which proposes a novel mechanism whereby the flow of cations in thin processes is restricted due to negatively charged membrane lipids which result in the formation of deep potential wells near the dipole heads. These wells restrict the flow of cations to “hopping” between adjacent wells as they transverse the process, and this surface retention of cations will be shown to give rise to the formation of potassium (K^+^) and sodium (Na^+^) microdomains at the PsC. We further propose that a K^+^ microdomain formed at the PsC, provides the driving force for the return of K^+^ to the extracellular space for uptake by the neurone, thereby preventing K^+^ undershoot. A slow decay of Na^+^ was also observed in our simulation after a period of glutamate stimulation which is in strong agreement with experimental observations. The pathological implications of microdomain formation during neuronal excitation are also discussed.

## Author summary

During periods of neuronal activity, ionic homeostasis in the surrounding extracellular space (ECS) is disturbed. To provide a healthy environment for continued neuronal function, excess ions such as potassium must be buffered away from the ECS; a vital supportive role provided by astrocyte cells. It has long been thought that astrocytes not only removed ions from the ECS but also transport them to other areas of the brain where their concentrations are lower. However, while our computational model simulations agree that astrocytes do remove these ions from the ECS they also show that these ions are mainly stored locally at the PsC to be returned to the ECS, thus restoring ionic homeostasis. Furthermore, we detail in this paper that this happens because of a previously overlooked biophysical phenomenon that is only dominant in thin astrocyte processes. The flow of these cations within thin processes is primarily by surface conduction where they experience the attraction of fixed negative charge at the membrane inner surface. This negative charge constrains cation movement along the surface and so their flow rate is restricted. Consequently, ions such as potassium that are released during neuronal excitation enter the PsC and are stored locally due to the low conductance pathway between the PsC and the astrocyte soma. Our simulations also show that this local build-up of K^+^ is returned to the ECS after the neuronal activity dies off which could potentially explain why K^+^ undershoot has not been observed; this result agrees with experimental observations. Moreover, the same mechanism can also explain the transient behaviour of Na^+^ ions whereby in thin processes a slow decay time constant is experimentally observed. These findings have important implications for the role of astrocytes in regulating neuronal excitability under physiological and pathological conditions, and therefore highlight the significance of the work presented in this paper.

## Introduction

Astroglia determine the architecture of neural tissue and maintain central nervous system (CNS) homeostasis [1–3]. Astrocytes are organised into functional syncytia that show anatomical specialisation [4, 5], which allow intercellular diffusion of ions, second messengers and metabolites. Astroglial membranes closely enwrap the majority of excitatory synapses in the CNS, forming astroglial cradles [6, 7]; a structure which facilitates synaptogenesis, synaptic maturation, synaptic transmission and synaptic extinction. Astroglial membranes are densely packed with transporters and ion pumps that maintain molecular homeostasis in the synaptic cleft and in the brain interstitium [8–11]. Furthermore, astrocytes maintain the homeostasis of many neurotransmitters and neuromodulators and supply neurones with glutamine, an essential precursor for the synthesis of glutamate and gamma-Aminobutric acid (GABA), the main excitatory and inhibitory neurotransmitters respectively [12–15].

K^+^ homeostasis is a canonical function of astroglia proposed in the mid-1960s; both energy dependent Na^+^/K^+^ATPase (NKA) and passive (inward rectifier K^+^ channels) pathways were considered as molecular mechanisms [9,16,17]. Subsequently the Na^+^/K^+^/Cl^-^ transporter NKCC1 was suggested to participate in K^+^ buffering, especially at high (pathological) K^+^ concentrations [10, 18, 19]. The local K^+^ uptake is supposedly supported by spatial K^+^ buffering (K^+^ diffusion through gap junctions from regions of elevated [K^+^] to regions of lower [K^+^]).

Under physiological conditions, however, the main pathway for K^+^ influx is associated with NKA, whereas K_ir_4.1 inward rectifying channels mediate K^+^ efflux which is needed to restore K^+^ gradients in neuronal compartments [10, 18, 19]. These observations are consistent with astrocytic K^+^ being re-released via K_ir_4.1 channels at distal synapses after distribution in the astrocytic functional syncytium via gap junctions [18]. However, in our paper we are dealing with K^+^ microdomains at the PsC, due to a low conductance pathway between the PsC and astrocyte soma, causing a significant increase in [K^+^]_PsC_ which returns to baseline level via K^+^ leak and K_ir_4.1 channels post neural excitation. Astroglial homeostatic function is, to a large extent, controlled by transmembrane Na^+^ gradients and is regulated by cytoplasmic Na^+^ signals [20, 21]. Dynamic fluctuations of [Na^+^]_i_ affect Na^+^-dependent transporters and associated molecular cascades that link Na^+^ dynamics to homeostasis of K^+^ and neurotransmitters [20, 21]. Astroglial Na^+^ signals are spatially heterogeneous with the existence of Na^+^ microdomains; these Na^+^ signals may also propagate through astroglial syncytia via gap junctions [22]. The main pathway for astroglial Na^+^ entry occurs through the excitatory amino acid transporters 1 and 2 (EAAT 1/2) [15]. Glutamate transport is powered by transmembrane ion gradients where 3 Na^+^ and 1 H^+^ ions are exchanged for one K^+^ ion, hence the Na^+^/glutamate transporter generates a net inward Na^+^ current [23, 24]. In addition, glutamate opens astroglial ionotropic receptors (AMPA and NMDA receptors) and activates (indirectly through store-operated Ca^+^ influx pathway) TRPC channels which further contribute to stimulus-dependent Na^+^ entry [25–28]. Glutamate transporters co-localise with sodium-calcium exchanger (NCX) that couple Na^+^ and Ca^2+^ signalling [29, 30].

The existence of astroglial ionic microdomains [21, 31, 32] at the PsC indicates that there must be mechanisms that slow ionic diffusion along the processes. A candidate for this mechanism is associated with fixed negative charges existing in cell membranes. Previous experiments [33] had found localised fixed negative charges in the membranes of neurones and glia. Biological membranes consist of a continuous bilayer of lipid molecules in which membrane proteins are embedded. These bilayers are filled with polar and non-polar portions in their structure (amphipathic) [34]. All eukaryotic membranes are also asymmetric such that biophysical properties differ between the intracellular and extracellular surfaces; this asymmetry is necessary for many key cellular processes including cell fusion and cell clearance [35].

As astrocyte processes are very thin we hypothesise that this effect will have a dominant role in restricting cation conduction along the membrane. Specifically, we propose that cation retention in the potential wells requires that cations must hop from well to well as they move along the thin astrocyte process and therefore, this hopping effect serves to semi-isolate the astrocytic perisynaptic cradle (PsC) from the astrocytic main body. In this paper we show, for the first time, that this slow leakage of cations could explain the formation of K^+^ and Na^+^ microdomains at the PsC which points to a new theory for K^+^ clearance. For example, it is widely reported [9, 36] that excitatory presynaptic neurones release K^+^ into the extracellular space (ECS) which is subsequently cleared at the PsC and buffered away through diffusion to the main astrocyte body. However, we propose that the flow of K^+^ away from the PsC is not volume diffusion limited, but rather is restricted due to the hopping effect along the process, and therefore a K^+^ microdomain forms at the PsC during sustained presynaptic neuronal excitation. Furthermore, we will show through mathematical modelling, that the formation of a K^+^ microdomain at the PsC may very well be advantageous as it facilitates a low energy return pathway for K^+^ to the ECS, after neuronal excitation has ceased. Additionally, cation retention along thin processes may also affect homeostasis for Na^+^ ions as they also carry a positive charge. It has been shown in other work [23] that the decay rate of Na^+^ following a sustained level of glutamate uptake (in the absence of extra-cellular K^+^ change) through EAAT1/2 is in the order of seconds. We will also show in this paper that this could potentially result from the restricted flow rate of Na^+^ ions along thin processes which results in the formation of a Na^+^ microdomain at the PsC that can only be removed by the NKA, reversal of NCX and other transporters.

## Models

**Fig 1:**
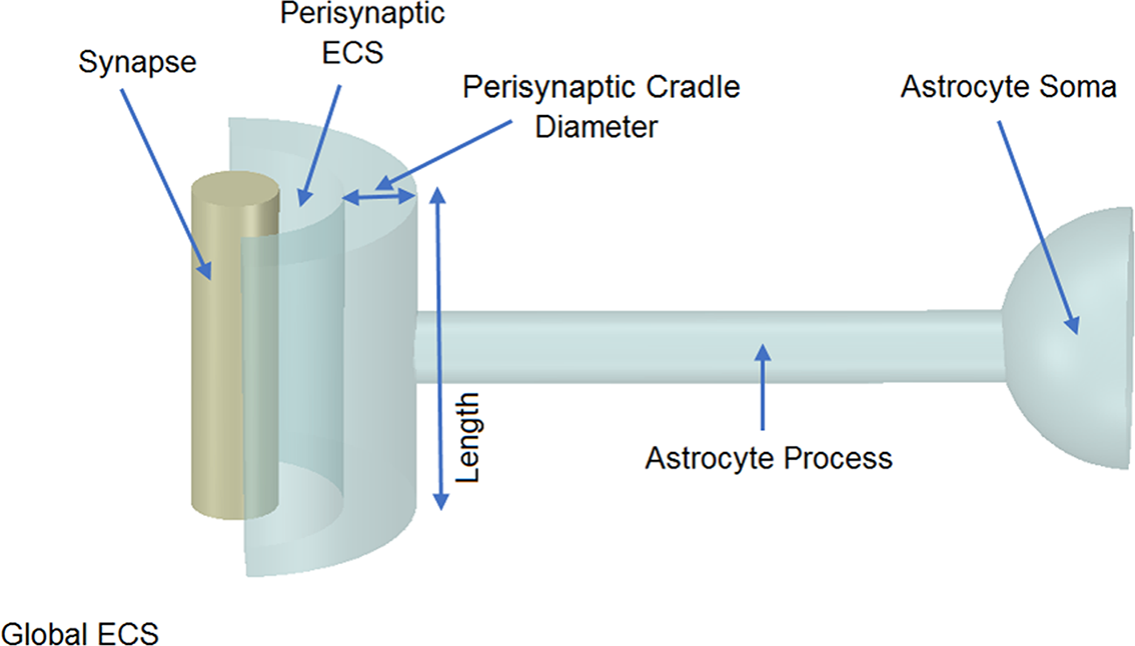
Model morphology. The model consists of a single synapse enwrapped by a single astrocyte. In total there are six compartments, 1) Global Extracellular Space (GECS), 2) Synapse, 3) Perisynaptic Extracellular Space (PsECS), 4) Perisynaptic Cradle, 5) Process, and 6) Astrocyte Soma. Each compartment is modelled as a cylindrical structure except the GECS and soma, which are not given dimensions because ionic concentrations remain constant within these compartments.

To explore the effects of cation retention in thin astrocytic process, a multi-compartmental model was developed consisting of a single synapse surrounded by an astrocytic PsC. Previous imaging studies [37–40] have highlighted the detailed neural/astroglial biological morphology which is comprised of extremely complex geometrical structures. To overcome the computation complexities of modelling the morphology of a synapse, where the influx/efflux of ions is 3-dimensional, and at the same time capturing the cradle-like structure [38] formed by the PsC around a synapse, we compartmentalise regions of interest using cylindrical arrangements, as shown in Fig 1.

Since the PsC does not completely enwrap the synapse, ions are free to diffuse from the PsECS into the GESC. Note that the ionic concentrations within the astrocyte soma and GECS are held constant but because of electro-diffusion between the GECS and PsECS, the concentration gradient local to the K_ir_ and other channels/pumps will vary. In the absence of data regarding the morphology of the GECS we adopted this approach as we need to have a reference point with fixed baseline ionic concentration levels and resting potential for our model.

Table 1 provides a reference to all morphological values where the diameter of the astrocyte process in this model was 100 nm [41]. From other work [42], both the half cylinder cradle inner diameter and height were assigned the value of 300 nm, the cradle thickness 100 nm and the perisynaptic ESC 30nm. All surface areas and volumes were calculated using these dimensions.

**Table 1:** Astrocyte morphology.

Parameter	Value	Units	Description	Ref
**Lengths:**				
**d**_**IPS**_	300 × 10^−9^	m	Perisynaptic internal diameter	[42]
**d**_**EPS**_	500 × 10^−9^	m	Perisynaptic external diameter	[42]
**r**_**IPS**_	150 × 10^−9^	m	Perisynaptic internal radius	[42]
**r**_**EPS**_	250 × 10^−9^	m	Perisynaptic external radius	[42]
**l**_**PS**_	300 × 10^−9^	m	Perisynaptic length	[42]
**d**_**P**_	100 × 10^−9^	m	Process diameter	[41]
**r**_**P**_	50 × 10^−9^	m	Process radius	[41]
**l**_**P**_	25 × 10^−6^	m	Process length	
**d**_**Syn**_	270 × 10^−9^	m	Synapse diameter	
**r**_**Syn**_	135 × 10^−9^	m	Synapse radius	
**l**_**syn**_	300 × 10^−9^	m	Synapse length	
**Areas:**				
**CSA**_**PS**_	3.5343 × 10^−14^	m^2^	Perisynaptic cross sectional area	
**SA**_**PS**_	1.4137 × 10^−13^	m^2^	Perisynaptic surface area	
**CSA**_**P**_	7.854 × 10^−15^	m^2^	Process cross sectional area	
**SA**_**P**_	7.854 × 10^−12^	m^2^	Process surface area	
**CSA**_**Syn**_	2.8628 × 10^−14^	m^2^	Synapse cross sectional area	
**SA**_**Syn**_	1.2723 × 10^−13^	m^2^	Synapse surface area	
**SA**_**PsECS-GECS**_	1.5715 × 10^−14^	m^2^	Surface area between PsECS and GECS	
**Volumes:**				
**Vol**_**PS**_	1.8850 × 10^−17^	L	Perisynaptic volume	
**Vol**_**P**_	1.9635 × 10^−16^	L	Process volume	
**Vol**_**Syn**_	8.5883 × 10^−16^	L	Synapse volume	
**Vol**_**PsECS**_	2.0145 × 10^−18^	L	Perisynaptic ECS volume	

**Fig 2:**
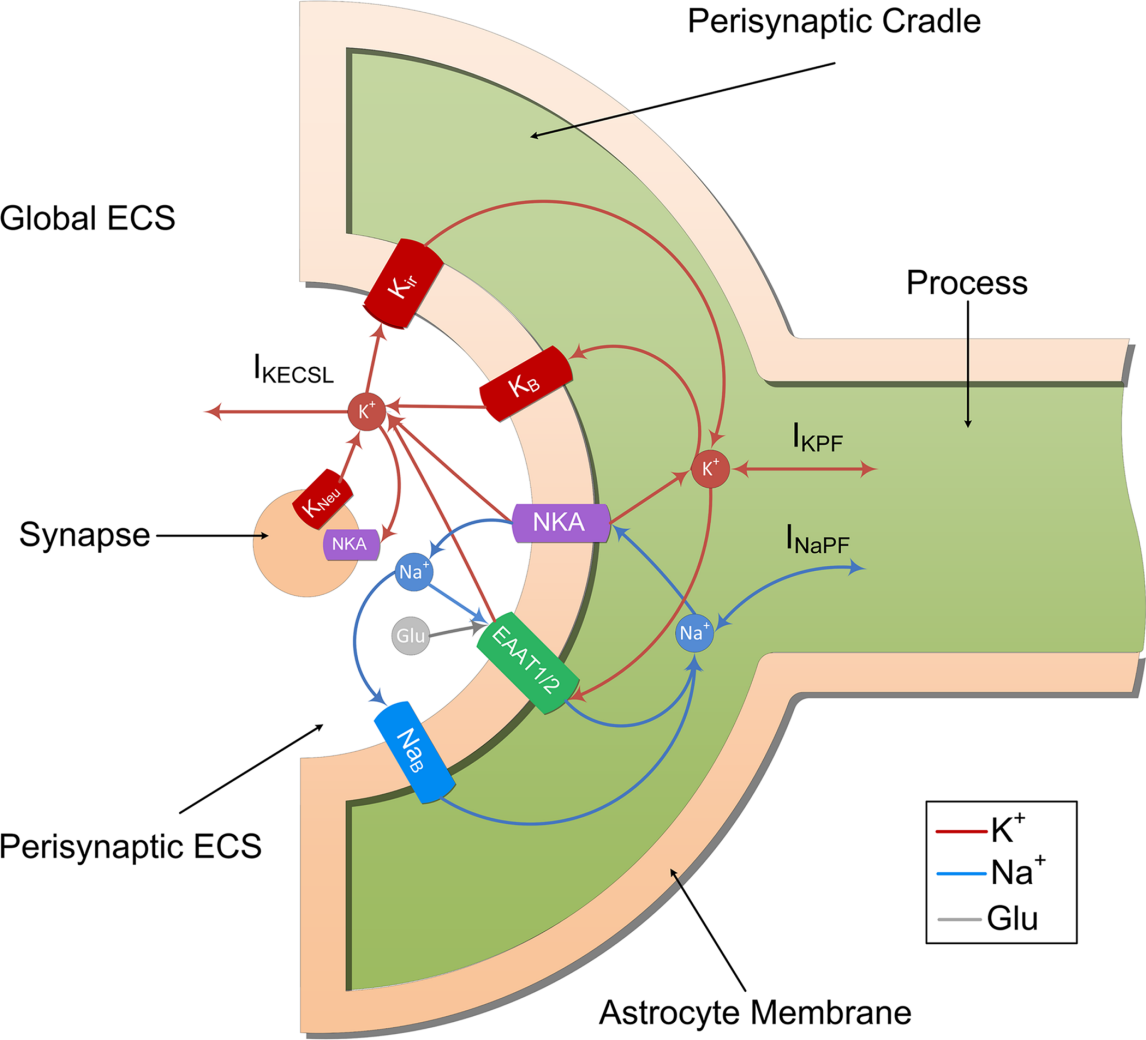
Ion transport machinery of the perisynaptic cradle and synapse.

The synapse and PsC contain various ionic channels, exchangers and pumps to provide homeostasis and dynamic exchange of ions between the two cells and extracellular space and are schematically shown in Fig 2 (Note: this diagram does not show the process connection to the astrocyte soma). As we are only considering Na^+^ and K^+^ dynamics at the PsC our model does not include both the Na-K-Cl cotransporter (NKCC1) and the NCX exchanger; these transporters depend on chloride and calcium respectively, both of which are not investigated in the current model. Furthermore, NKCC1 is not the dominant uptake mechanism for K^+^ for ECS K^+^ elevation under 10 mM [43].

The complete model contains 7 transport mechanisms which move ions across the membranes. The neuronal model exchanges K^+^ and Na^+^ with the PsECS via a potassium channel (K_Neu_) and a sodium potassium pump (NKA_Neu_), Note: neuronal NKA K^+^ binding site is completely saturated, so the only ion that activates neuronal NKA is cytosolic Na^+^. The astrocyte exchanges Na^+^ and K^+^ with the PsECS via a background sodium transport (Na_B_), background potassium transport (K_B_), potassium inwardly rectifying channel (K_ir_), sodium-potassium-ATPase (NKA), and a glutamate-sodium-potassium cotransporter (EAAT1/2). I_KPF_ and I_NaPF_ model the current flow of K^+^ and Na^+^ along the process to the soma. I_KECSL_ models the K^+^ current generated by K^+^ leaking from the PsECS to the GECS. The mathematical descriptions of these components are now described in detail.

### Astrocyte model

#### Ion concentrations and membrane voltage

The perisynaptic model comprises five compartments, PsC, perisynaptic extracellular space (PsECS), global extracellular space (GECS), and the astrocyte process and soma. Each of these compartments comprises of three ionic concentrations, K^+^, Na^+^ and Glu. All channels sensitive to these ions reside on the PsC while the astrocyte process is modelled as a long thin cylindrical channel that restricts the flow of cations within the channel due to ion retention (see later). The kinetic equations for the changes of ionic concentration of each of these ions is given below. Note: z_x_FVol_y_ is used to convert the total ionic current of ion x into a concentration for the volume y, where z_x_ is the valency of ion x, F is Faradays constant and Vol_y_ is the volume of compartment y. All initial conditions and parameters for this model are described in Table 2 and Table 3 respectively. The change in PsC K^+^ concentration ([K^+^]_PsC_) in the PsC is given by:

$$\frac{{d[K^{+}]}_{PsC}}{dt}=-\left(\frac{I_{Kir}+I_{KNKA}+I_{KEAAT}+I_{KPF}}{z_{K}F{Vol}_{PsC}}\right)$$ (1)

where I_Kir_ is the K_ir_ channel current, I_KNKA_ is the K^+^ current through the NKA, I_KEAAT_ is the K^+^ current created by the glutamate transporter, and *I*_*KPF*_ is the K^+^ current flowing along the astrocyte process. K^+^ changes in the PsECS ([K^+^]_PsECS_) is given by:

$$\frac{{d[K^{+}]}_{PsECS}}{dt}=-\left(\frac{I_{KECSL}+I_{KNeu}-I_{Km}}{z_{K}F{Vol}_{PsECS}}\right)$$ (2)

where I_KECSL_ is current due to K^+^ leakage from the PsECS to the GECS, I_KNeu_ is the K^+^ current from the neurone and I_Km_ is the total K^+^ current flowing through the astrocyte membrane. K^+^ is held constant in the GECS and astrocyte soma compartments.

**Table 2:** Astrocyte model variables.

Variable	Initial Value	Units	Description	Ref
**V**_**A**_	-0.09	V	Astrocyte Membrane potential	
**[K**^**+**^**]**_**PsC**_	0.1	M	K^+^ concentration in the perisynaptic cradle	[46]
**[Na**^**+**^**]**_**PsC**_	0.015	M	Na^+^ concentration in the perisynaptic cradle	[46]
**[K**^**+**^**]**_**PsECS**_	0.003	M	Perisynaptic extracellular K^+^ concentration	[46]
**[Glu]**_**ECS**_	1 × 10^−6^	M	Perisynaptic extracellular Glutamate concentration	[52]

**Table 3:** Astrocyte model parameters.

Parameter	Value	Units	Description	Ref
**V**_**m**_	-0.09	V	Astrocyte resting membrane potential	
**φ**_**w**_	0.267	eV	Well activation energy	[51]
**k**_**B**_	1.38 × 10^−23^	J/K	Boltzmann constant	
**R**	8.31	J/mol/K	Gas constant	
**T**	310	K	Temperature	
**F**	96485	C/mol	Faraday constant	
**Q**	1.6022 × 10^−19^	C	Coulomb	
**C**_**m**_	0.01	F/m^2^	Membrane capacitance	[46]
**g**_**Kir**_	144	S/m^2^	K_ir_ channel conductance	[36]
**g**_**K**_	16.9131	S/m^2^	K^+^ background transport conductance	
**g**_**Na**_	0.4293	S/m^2^	Na^+^ background transport conductance	
**K**_**K**_	0.018	S/m	K^+^ Poole-Frenkel channel constant	
**K**_**Na**_	0.018	S/m	Na^+^ Poole-Frenkel channel constant	
**PNKA**_**max**_	0.1 × 10^−5^	mol/m^2^	Maximum NKA-ATPase Pump Rate	[46]
**K**_**Nai**_	1.5 × 10^−3^	M	Na^+^ threshold for NKA-ATPase	
**K**_**KE**_	10 × 10^−3^	M	K^+^ threshold for NKA-ATPase	
**z**_**K**_	1		K^+^ Valency	
**z**_**Na**_	1		Na^+^ Valency	
**z**_**Glu**_	-1		Glu Valency	
**[Glu]**_**PsC**_	1.5 × 10^−3^	M	Glu concentration in the perisynaptic cradle	[53]
**[H**^**+**^**]**_**PsC**_	60 × 10^−9^	M	H^+^ Concentration in the perisynaptic cradle	
**[K**^**+**^**]**_**AS**_	0.1	M	K^+^ Concentration in the astrocyte soma	
**[Na**^**+**^**]**_**AS**_	0.015	M	Na^+^ Concentration in the astrocyte soma	[46]
**[H**^**+**^**]**_**PsECS**_	40 × 10^−9^	M	Perisynaptic extracellular H^+^ concentration	
**[Na**^**+**^**]**_**PsECS**_	0.145	M	Perisynaptic extracellular Na^+^ concentration	[46]
**[K**^**+**^**]**_**GECS**_	0.003	M	Perisynaptic global ECS K^+^ concentration	[46]
**[Na**^**+**^**]**_**GECS**_	0.145	M	Perisynaptic global ECS Na^+^ concentration	[46]
**ε**_**0**_	8.85 × 10^−12^	F/m	Vacuum permittivity	
**ε**_**r**_	0.82	F/m	Relative permittivity of brain tissue	[54]
**g**_**ECS**_	3.3	S/m^2^	Perisynaptic ECS leak conductance	
**α**_**EAAT**_	0.0032	A/m^2^	Glutamate transport fitting parameter	
**β**_**EAAT**_	0.0288	V^-1^	Glutamate transport fitting parameter	
**r**_**g**_	5 × 10^−7^	M^-1^	Slope of glutamate uptake	
**s**_**g**_	9 ×10^−6^	M	Threshold for glutamate uptake	

Changes in the PsC Na^+^ concentration ([Na^+^]_PsC_) is given by:

$$\frac{{d[{Na}^{+}]}_{PsC}}{dt}=-\left(\frac{I_{NaB}+I_{NaNKA}+I_{NaEAAT}+I_{NaPF}}{z_{Na}F{Vol}_{PsC}}\right)$$ (3)

where I_NaB_ is a current due to Na^+^ influx across the membrane via Na^+^ permeable ion channels, this is referred to as background Na^+^ channel, I_NaNKA_ is the Na^+^ dependent current component of the NKA, I_NaEAAT_ is the Na^+^ current component of the glutamate transporter, and I_NaPF_ is the Na^+^ current flowing in the astrocyte process. [Na^+^] changes in all other compartments are not considered and remain constant.

Changes in the PsECS Glu concentration ([Glu]_PsECS_) is given by:

$$\frac{{d[Glu]}_{PsECS}}{dt}=-\left(\frac{-I_{GluEAAT}}{z_{Glu}F{Vol}_{PsECS}}\right)$$ (4)

where I_GluEAAT_ is the Glu current component of the glutamate transporter. [Glu] changes in all other compartments are not considered and remain constant. Note: In this work we do not fully model neurone/astrocyte glutamate transport i.e. Glu influx/efflux pathways, therefore, to prevent [Glu]_PsECS_ from draining completely via EAAT1/2 activity which is always active in the inward direction, we simply hold [Glu]_PsECS_ at its initial value when it falls below its initial value.

The membrane voltage of the astrocyte varies according to:

$$C_{m}\frac{dV_{A}}{dt}=I_{Km}+I_{Nam}$$ (5)

where C_m_ is the membrane capacitance and I_Km_ and I_Nam_ and is the total current flow across the perisynaptic membrane ion channels and are given by:

$$I_{Km}=I_{KB}+I_{Kir}+I_{KNKA}+I_{KEAAT}$$ (6)

$$I_{Nam}=I_{NaB}+I_{NaNKA}+I_{NaEAAT}$$ (7)

#### Inward rectifying potassium channel (Kir)

The K_ir_ channel is a voltage dependent ion channel with a high affinity to K^+^. The current flow through this channel is given by [36]:

$$I_{KIR}=g_{K}\sqrt{{\left[K^{+}\right]}_{PsECS}}(V_{A}-E_{K}){SA}_{PsC}$$ (8)

where g_K_ is the channel conductance, V_A_ is the astrocyte membrane voltage, SA_PsC_ is the surface area of the perisynaptic membrane and E_K_ is the Nernst potential of the channel which is set at 25mV.

#### Background ion channels (Na_B_ / K_B_)

In this model, there are two separate background ion channels for Na^+^ and K^+^. These channels use the electrochemical gradient between the PsC and ECS resulting in an influx of Na^+^ and an efflux of K^+^ under normal physiological conditions [21,44]. They were modelled as a simple passive electrochemical gradient dependent channel given by [45]:

$$I_{iB}=g_{i}(V_{A}-E_{i}){SA}_{PsC}$$ (9)

where i is the ion under consideration, g_i_ is the channel conductance. Note: the value of g_i_ is chosen in such a way that at steady state I_im_ = 0. V_A_ is the astrocyte membrane voltage, SA_PsC_ is the surface area of the perisynaptic membrane, and E_i_ is the channel Nernst potential and is given by:

$$E_{i}=\frac{RT}{F}ln\left(\frac{{\left[i^{+}\right]}_{PsECS}}{{\left[i^{+}\right]}_{PsC}}\right)$$ (10)

#### Sodium potassium pump (NKA)

The NKA exchanges intracellular Na^+^ for extracellular K^+^ against the gradient of both ions and has a stoichiometry of 3:2. The K^+^ current component of the pump is given by [46]:

$$I_{KNKA}=2\;F\;P_{NKAmax}\frac{{\left[{Na}^{+}\right]}_{PsC}^{1.5}}{{\left[{Na}^{+}\right]}_{PsC}^{1.5}+K_{Nai}^{1.5}}\;\frac{{\left[K^{+}\right]}_{PsECS}}{{\left[K^{+}\right]}_{PsECS}+K_{KE}}\;{SA}_{PsC}$$ (11)

and the Na^+^ current component of the pump is given by:

$$I_{NaNKA}=-3\;F\;P_{NKAmax}\frac{{\left[{Na}^{+}\right]}_{PsC}^{1.5}}{{\left[{Na}^{+}\right]}_{PsC}^{1.5}+K_{Nai}^{1.5}}\;\frac{{\left[K^{+}\right]}_{PsECS}}{{\left[K^{+}\right]}_{PsECS}+K_{KE}}\;{SA}_{PsC}$$ (12)

where P_NKAmax_ is the NKA maximum pump rate, K_Nai_ is the Na^+^ threshold of the pump, and K_KE_ is the K^+^ threshold of the pump.

#### Glutamate transporter (EAAT1/2)

The EAAT1/2 is responsible for the uptake of extracellular glutamate from the PsECS. Among other ions, it countertransports 1 K^+^ and cotransports 3 Na^+^ with every glutamate ion [47]. To model this stoichiometry, we adjust the model presented by [48] which describes glutamate uptake by the astrocyte and has a constant transport rate. However, in our model the maximum glutamate transport current (I_mg_) of the cotransporter is dependent on intra- and extra- cellular levels of K^+^, Na^+^, H^+^, and glutamate. The K^+^ current component of the transporter is given by:

$$I_{KEAAT}=-\frac{I_{mg}}{\left(1-exp(r_{g}s_{g}-r_{g}[{Glu]}_{PsECS})\right)}\;{SA}_{PsC}$$ (13)

the Glu current component of the transporter is the same magnitude as the K^+^ component but in the opposite direction and is given by:

$$I_{GluEAAT}=-I_{KEAAT}\;{SA}_{PsC}$$ (14)

and the Na^+^ current component is given by:

$$I_{NaEAAT}=3\frac{I_{mg}}{\left(1-exp(r_{g}s_{g}-r_{g}{\left[Glu\right]}_{PsECS})\right)}\;{SA}_{PsC}$$ (15)

where I_mg_ is the maximum uptake current of glutamate, r_g_ is the slope of the glutamate uptake, s_g_ is the threshold of the glutamate uptake. I_mg_ is given by:

$$I_{mg}=\frac{1}{6}\left(-\alpha_{EAAT}(exp({-\beta }_{EAAT}(V_{A}-V_{rev}))-1)\right)$$ (16)

The constants α_EAAT_ and β_EAAT_ are fitting parameters, V_A_ is the astrocyte membrane potential and V_rev_ is the EAAT reversal potential, i.e. the driving force.

V_rev_ is dependent on the intra- and extra- cellular concentrations of K^+^, Na^+^, H^+^, and glutamate and is given by:

$$V_{rev}=\frac{RT}{2F}ln\left({\left(\frac{{\left[{Na}^{+}\right]}_{PsECS}}{{\left[{Na}^{+}\right]}_{PsC}}\right)}^{3}\frac{{\left[K^{+}\right]}_{PsC}}{{\left[K^{+}\right]}_{PsECS}}\frac{{\left[H^{+}\right]}_{PsECS}}{{\left[H^{+}\right]}_{PsC}}\frac{{\left[{Glu}^{+}\right]}_{PsECS}}{{\left[{Glu}^{+}\right]}_{PsC}}\right)$$ (17)

Considering the electrophysiology of the EAAT1/2 reversal potential, we would point out that this potential is defined mainly by glutamate because glutamate rises by 3 orders of magnitude in comparison to Na^+^, H^+^, K^+^ and therefore cannot reverse. Note, as no account is taken of intracellular glutamate, no change in PsC glutamate is calculated and therefore remains constant.

### Astrocyte process ionic transport model

Both sides of the bilayer phospholipid membrane surface are negatively charged, however, it has been shown that the distribution of charge is non-uniform at the atomic scale [49]. This non-uniformity gives rise to a potential profile including deep potential wells that can trap ions close to the membrane. Basic quantum mechanics show that regardless of the depth of a well it will have at least one state that can trap the travelling ions. The main effect of the depth of the well is the energy needed for the ion to continue its motion. This means that the ions cannot move easily along the membrane and must “hop” from well to well [49, 50]. Charge hopping transport has been extensively studied in dielectric and semiconductor materials. The present case is particularly complex because of the presence of mobile cations in the cytoplasm, causing the formation of an electrical double layer near the interface between the cytoplasm and the membrane. The negative fixed charge present on the membrane is due to phosphatidylserine (PS) which is composed of phosphatidic acid (PA), with the negatively charged phosphate group attached to the amino acid serine at the hydroxyl end. The associated negative charge will cause cations to move towards the membrane creating a dynamic space charge distribution in the cytoplasm as cations move from one well to an adjacent one; that is, there will be a net movement of cation ions due to the electric field along the length of the process. This leads to a complex and dynamic 3D potential distribution, which is beyond the scope of this paper. However, in order to investigate the interplay between the retention of cations along the process length and the formations of microdomain at the PsC we draw on a well-established charge hopping model for electrons in dielectrics containing Coulombic trapping centres [51]. In our case, the membrane fixed charge and associated potential wells are deemed analogous to Coulombic trapping centres. The current flow I_iPF_ through the thin process is then represented as:

$$I_{iPF}=K_{i}\frac{{V_{A}-V_{m}-V}_{r}}{l}exp\left[-\frac{Q_{i}(\varphi_{w-}\sqrt{\frac{Q_{i}(V_{A}-V_{m}-V_{r})}{l\pi \epsilon }})}{k_{B}T}\right]\;{CSA}_{P}$$ (18)

where K is a constant which represents mobility and concentration of mobile ions, V_m_ is the resting membrane potential of the astrocyte, φ_w_ is the well activation energy or potential barrier to ion flow. It has been shown that φ_w_ lies typically in the range (1–20) × k_B_T [51]. and is initially taken as 10 k_B_T but the effect of changing φ_w_ will be considered later; l is the length of the process, Q is the charge on a single ion taken as the charge on an electron, T is the absolute temperature, CSA_P_ is the cross-sectional area of the process, ϵ is the dynamic permittivity and is given by ϵ = ϵ_0_ ϵ_r_, where ϵ_0_ is the absolute permittivity and ϵ_r_ is the relative permittivity of the cytoplasm, and k_B_ is the Boltzmann constant. Note that the potential across the length of the process is assumed linear in this formulation; that is the lateral electric field is constant. The square root term in the argument of the exponential term of Eq 16 represents the field-dependent lowering of the activation energy, φ_w_. The ‘trapping centres’ are assumed to be spaced relatively widely such that their potential distributions do not overlap.

**Fig 3:**
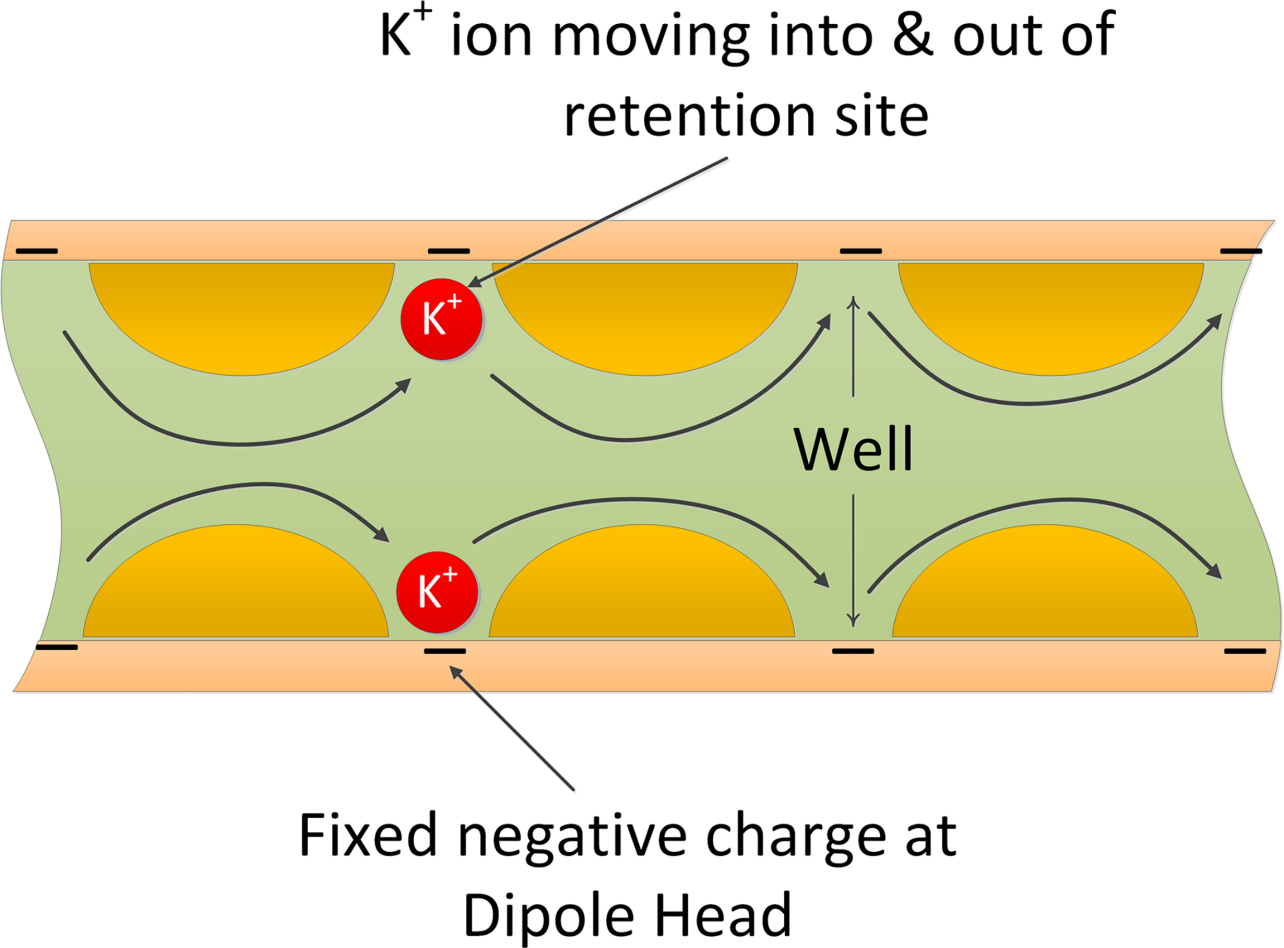
Cross section of a thin process showing charge hopping transport mechanism. The fixed negative charge on the inner membrane surface attracts positively charged cations into wells causing hopping from well to well along the length of the process.

The concentrations of K^+^ and Na^+^ in the astrocyte soma are held constant but will be continuously changing at the PsC thus establishing a dynamic concentration gradient associated with these cations. Consequently, we formulate a Nernst-like reversal potential for Na^+^ and K^+^ between the astrocyte soma (AS) and the PsC as:

$$V_{r}=\frac{RT}{F}ln\left(\frac{{\left[i\right]}_{AS}}{{\left[i\right]}_{PsC}}\right)$$ (19)

where i is the ion under consideration. A schematic of the hopping concept is shown in Fig 3.

### Leakage from perisynaptic ECS to global ECS

The diffusion of K^+^ between the PsECS and the GECS is modelled as a simple gradient controlled channel and is given by:

$$I_{KECSL}=g_{ECS}E_{ECS}{SA}_{ECSL}$$ (20)

where g_ECS_ is the conductance of the channel, SA_ECSL_ is the surface area between the PsECS and the GECS, and E_ECS_ is the Nernst like potential of the channel given by:

$$E_{ECS}=\frac{RT}{F}ln\left(\frac{{\left[K^{+}\right]}_{PsECS}}{{\left[K^{+}\right]}_{GECS}}\right)$$ (21)

### Neurone model

The neuronal model utilised in the work consists of the biophysical Hodgkin and Huxley (HH) type model (described in supplementary material S1 Text) [55] with the addition of NKA. All parameter values for the NKA can be found in Table 4.

**Table 4:** NKA parameters.

Parameter	Value	Units	Description
**P**_**NKAmaxNeu**_	-6.8017 × 10^−9^	mol/m^2^	Maximum NKA-ATPase Pump Rate
**K**_**NaiNeu**_	1.5 × 10^−3^	M	Na^+^ threshold for NKA-ATPase
**K**_**KENeu**_	10 × 10^−3^	M	K^+^ threshold for NKA-ATPase
**[Na**^**+**^**]**_**Syn**_	0.015	M	Na^+^ concentration in the synapse

Neurone potassium channel (K_Neu_). The HH model simulates current flow of K^+^ through a voltage gated channel, therefore the current flow of K^+^ from the neurone can be modelled as:

$$I_{KNeu}={-\;g}_{KNeu}n^{4}(V_{Neu}-E_{KNeu}){SA}_{Syn}$$ (22)

where g_KNeu_ is the maximum K^+^ channel conductance, E_KNeu_ is the reversal potential of the potassium channel, V_Neu_ is the membrane voltage of the neurone and SA_syn_ is the surface area of the synapse (neurone parameters described in supplementary material S1 Table).

Neurone sodium potassium pump (NKA_Neu_). Similar to the astrocytic NKA, the neuronal NKA exchanges intracellular Na^+^ for extracellular K^+^ against the gradient of both ions and has a stoichiometry of 3:2. The K^+^ current component of the pump is given by [46]:

$$I_{KNKANeu}=2\;F\;P_{NKAmaxNeu}\frac{{\left[{Na}^{+}\right]}_{Syn}^{1.5}}{{\left[{Na}^{+}\right]}_{Syn}^{1.5}+K_{NaiNeu}^{1.5}}\;\frac{{\left[K^{+}\right]}_{PsECS}}{{\left[K^{+}\right]}_{ECS}+K_{KENeu}}\;{SA}_{Syn}$$ (23)

where P_NKAmaxNeu_ is the NKA maximum pump rate, K_NaiNeu_ is the Na^+^ threshold of the pump, and K_KENeu_ is the K^+^ threshold of the pump. Since the neural model does not contain all ion channels necessary for homeostasis, Na^+^ changes due to the neuronal NKA pump both inside and outside the synapse are not taken into account. The value of P_NKAmaxNeu_ was chosen in such a way that I_KNKANeu_ = 0 at steady state.

## Results

In this section we test the validity of our hypothesis through simulations. The aim is to show that cation retention in thin processes leads to the formation of ionic microdomains at the PsC: Na^+^ microdomains were experimentally observed [23] and our hypothesis may very well explain their origin. Specifically, we show that a K^+^ microdomain formed at the PsC, provides the driving force for the return of K^+^ to the extracellular space for uptake by the neurone, thereby preventing K^+^ undershoot. We also show that our model can explain the slow decay of Na^+^ at the PsC after a period of glutamate stimulation, which is in strong agreement with experimental observations [23]. Finally, we use our model to predict the dynamic behaviour of ions under more physiological conditions whereby we simulate neuronal co-release of K^+^ and glutamate from the presynaptic terminal.

The simulation results presented in this section were obtained using Matlab 2015b 64 bit (Windows version) by Mathworks. The forward Euler method of integration was used for simulation with a fixed time step of Δ*t* = 10*μ*s.

### ECS K^+^ driven PsC K^+^ microdomain formation

**Fig 4:**
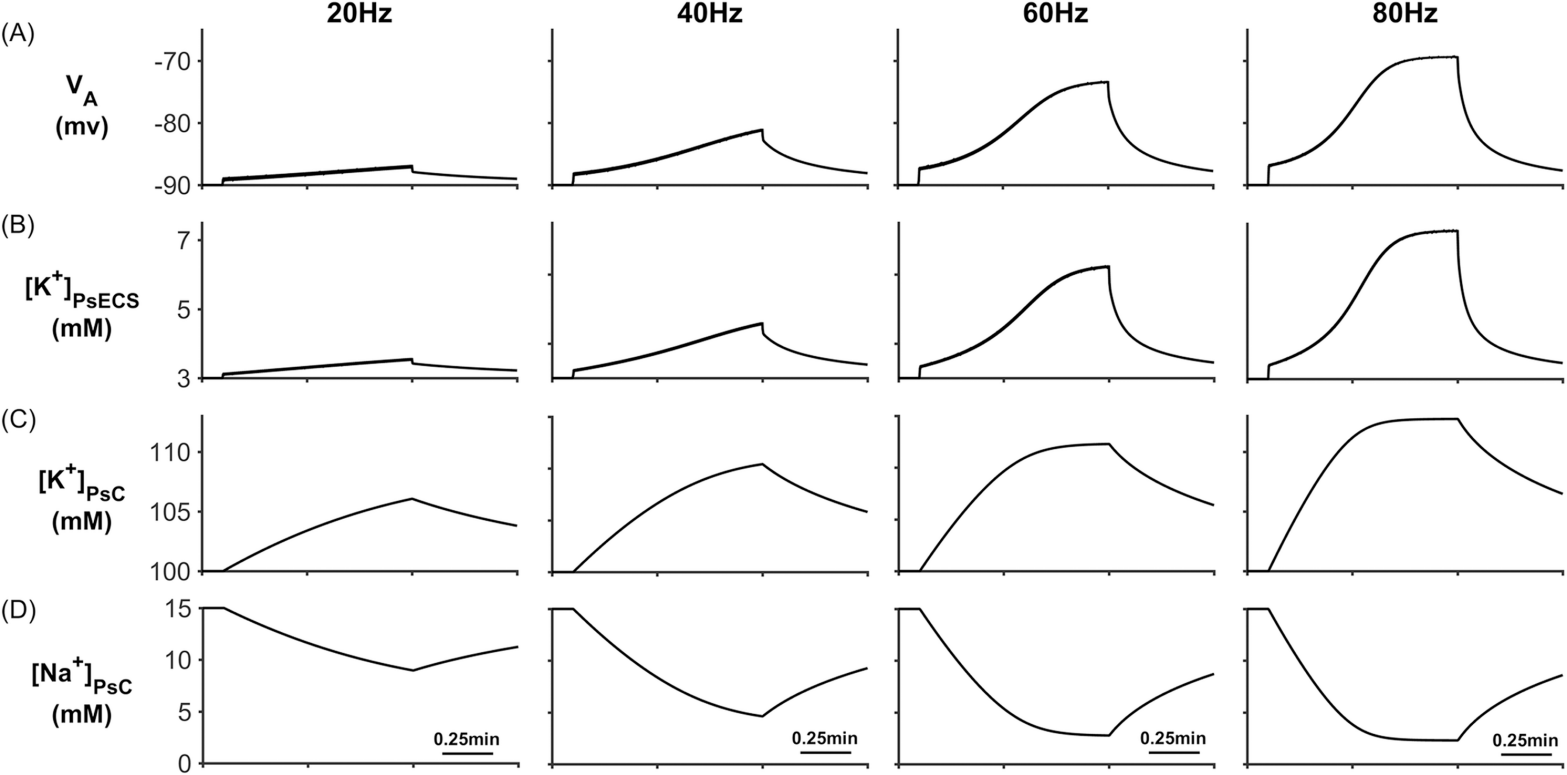
PsC membrane voltages and concentrations against time. (A) Astrocyte membrane voltage (V_A_). (B) [K^+^]_PsECS_. (C) [K^+^]_PsC._ transient. (D) [Na^+^]_PsC_ transient.

To explore how K^+^ retention in the astrocyte process gives rise to a K^+^ microdomain at the PsC and eliminates K^+^ undershoot, several simulations were carried out with the presynaptic neurone stimulated using external currents to produce firing rates of 20Hz, 40Hz, 60Hz and 80Hz. These firing rates are all within physiological frequencies of most cortical pyramidal neurones and fast spiking neurones. The neural stimulus has a duration of ~1 minute where the first 0.1 minute allows the model to reach a steady state condition and the stimulus ceases after 1min. Although this is a long period of time, it allowed an investigation into how extracellular and intracellular ionic concentrations would be affected during a sustained period of neural activity. For each simulation, PsECS [Glu] was held constant at the background level. Fig 4 describes [K^+^] and [Na^+^] dynamics for each of the 4 different stimuli where it can be seen that neuronal release of K^+^ into the PsECS leads to an increase in the astrocyte membrane voltage (V_A_ in Fig 4A) because of the change in ionic currents through the PsC membrane. It can also be seen that K^+^ steadily increases within the PsECS ([K^+^]_PsECS_ in Fig 4B) and after a period of ~0.8 minutes it approaches steady state at higher frequencies where the release rate of K^+^ by the presynaptic neurone equates to the clearance rate by NKA and K_B_ on both the PsC and the presynaptic terminal, and also K^+^ lost into the GECS. It is also worth noting that as the concentration of K^+^ increase in the PsC ([K^+^]_PsC_ in Fig 4C), the Na^+^ concentration with the PsC decrease due to efflux by NKA at the PsC ([Na^+^]_PsC_ in Fig 4D). Note: the astrocyte membrane voltage V_A_, [K^+^]_PsECS_ and [K^+^]_PsC_ all increase with the presynaptic neurone firing rate while [Na^+^]_PsC_ decreases.

**Fig 5:**
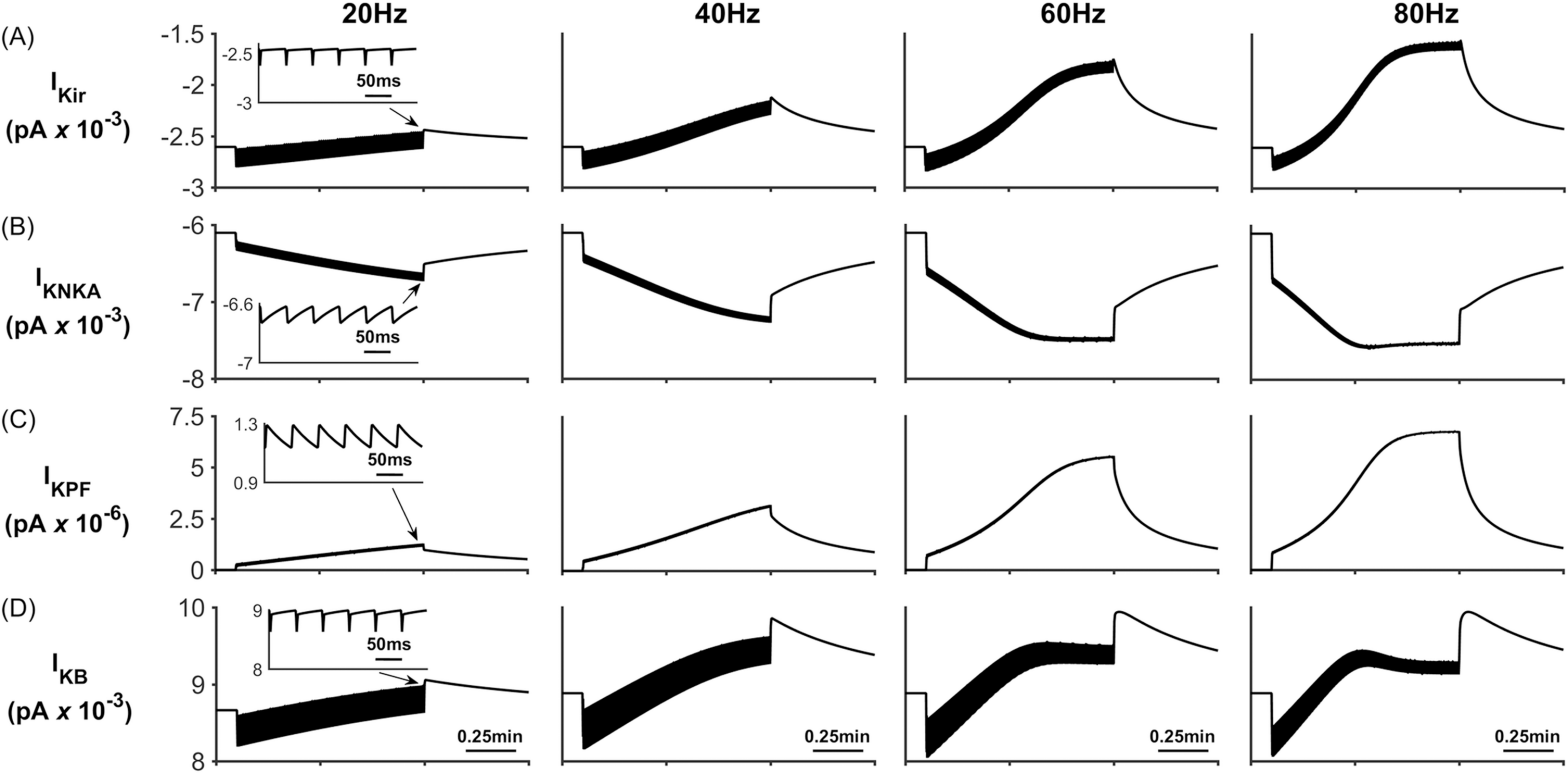
Perisynaptic K^+^ currents. (A) K^+^ K_ir_ current. (B) K^+^ NKA current. (C) K^+^ current along the process. (D) Background K^+^ current.

During neural activity, the NKA and K_ir_ channel currents are responsible for K^+^ uptake while the background K^+^ and KPF currents release K^+^ from the PsC. These currents can be seen in Fig 5 where Fig 5B shows that, contrary to the current thinking [56], the NKA is the dominant driving force for K^+^ uptake while K_ir_ channel (Fig 5A) is much less so for K^+^ clearance: furthermore, clearance by K_ir_ diminishes over time because the changes in the associated reversal potential due to the [K^+^]_PsC_ microdomain. Fig 5C shows that I_KPF_ is several orders of magnitude lower than I_Kir_ and therefore this slow leakage of K^+^ away from the PsC appears to be a plausible explanation for the emergence of a K^+^ microdomain. Note the saturation and subsequent fall off of I_KB_ at higher frequencies is a direct result of the K^+^ background reversal potential approaching V_A_. This is caused by the rapid build-up of K^+^ in the PsECS and cradle. The high frequency oscillatory behaviour which appears as a thickening of Fig 5A–5D is due to the astrocytic response to the pulsed nature of presynaptic neuronal K^+^ release. As the potassium in the PsECS fluctuates so does the astrocyte NKA pump and to a lesser extent the astrocyte membrane voltage. These fluctuations in the NKA and membrane voltage are also reflected in Na^+^ and K^+^ currents. Inserts in Fig 5A–5D, column 1, are used to show detail of astrocyte K^+^ current dynamics in response to neurone K^+^ release. Note: for clarity only the first column shows this detail as the dynamics for each current is similar for each of the stimulus frequencies.

**Fig 6:**
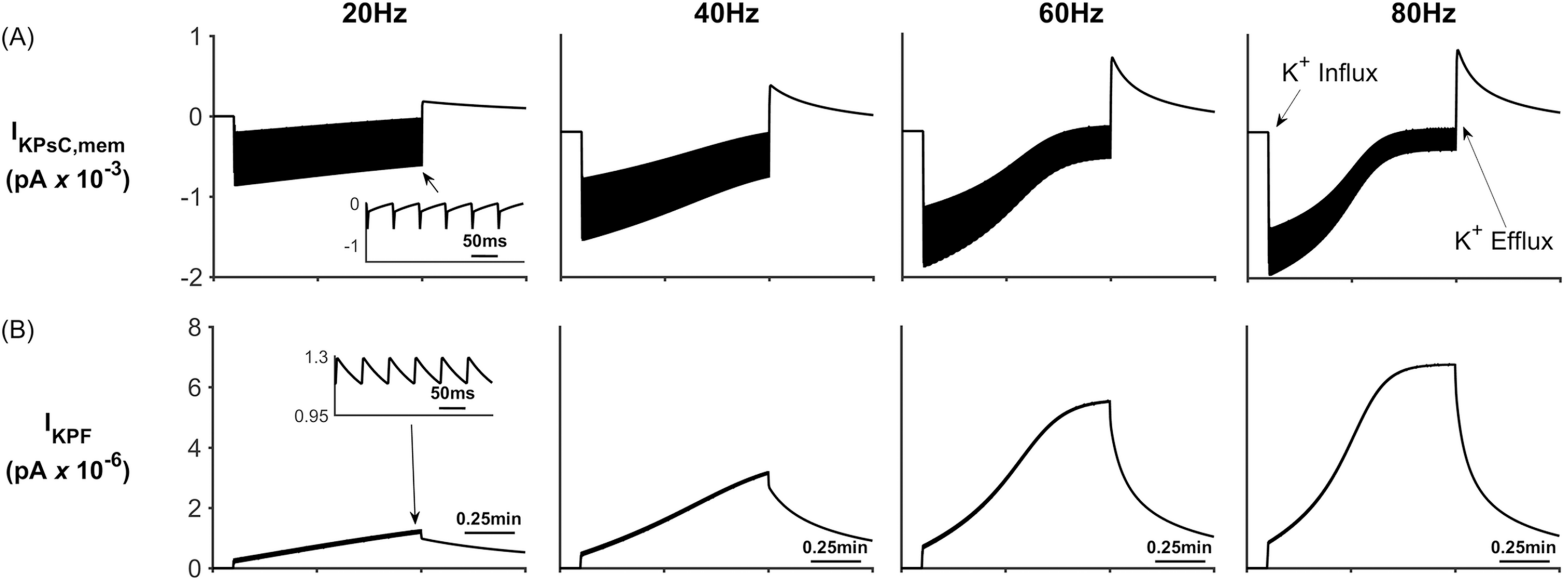
Total perisynaptic membrane K^+^ current and K^+^ process current. (A) The total K^+^ current flowing across the perisynaptic membrane (I_KPSC,mem_). During neural activity (Start = 0.1 min) K^+^ in the PsECS is removed by NKA and there is a net influx of K^+^. When neural activity stops (1min), K^+^ is released back into the PsECS mediated by the background K^+^ channel. This influx/efflux can be seen in A column 4. (B) K^+^ current flowing along the process (I_KPF_).

When the neurone stops releasing K^+^ (~ 1min) it quickly flows from the PsECS into the ECS which reduces the K^+^ gradient between the PsECS and PsC thereby reducing NKA pump rate, after which a net efflux of K^+^ takes place from the stored K^+^ in the associated microdomain. This points to a new theory whereby K^+^ microdomain formation during neuronal excitation (due to ion retention in the astrocyte process) provides the driver for the return of K^+^ to the PsECS, via background K^+^ leak and K_ir_4.1 channels, for uptake by the neurone. Fig 6A shows the net transfer of K^+^ across the perisynaptic membrane while Fig 6B shows the net current flow along the process (out of the perisynaptic cradle). During stimulation (0.1 min to 1min) it can be seen that there is a net transfer of K^+^ into the perisynaptic cradle across the membrane (Fig 6A). Since the current flowing along the process to the soma (Fig 6B) is 3 orders of magnitude smaller than the currents entering the cradle, there is a net build-up of K^+^: essentially a K^+^ microdomain forms because of the low conductance pathway from the cradle to the astrocyte soma. Furthermore, this microdomain allows the efflux of K^+^ from the PsC into the PsECS after neurone stimulation ceases. This can be seen as a spike like current in Fig 6A after 1min and is more pronounced in the 80Hz simulation.

**Fig 7:**
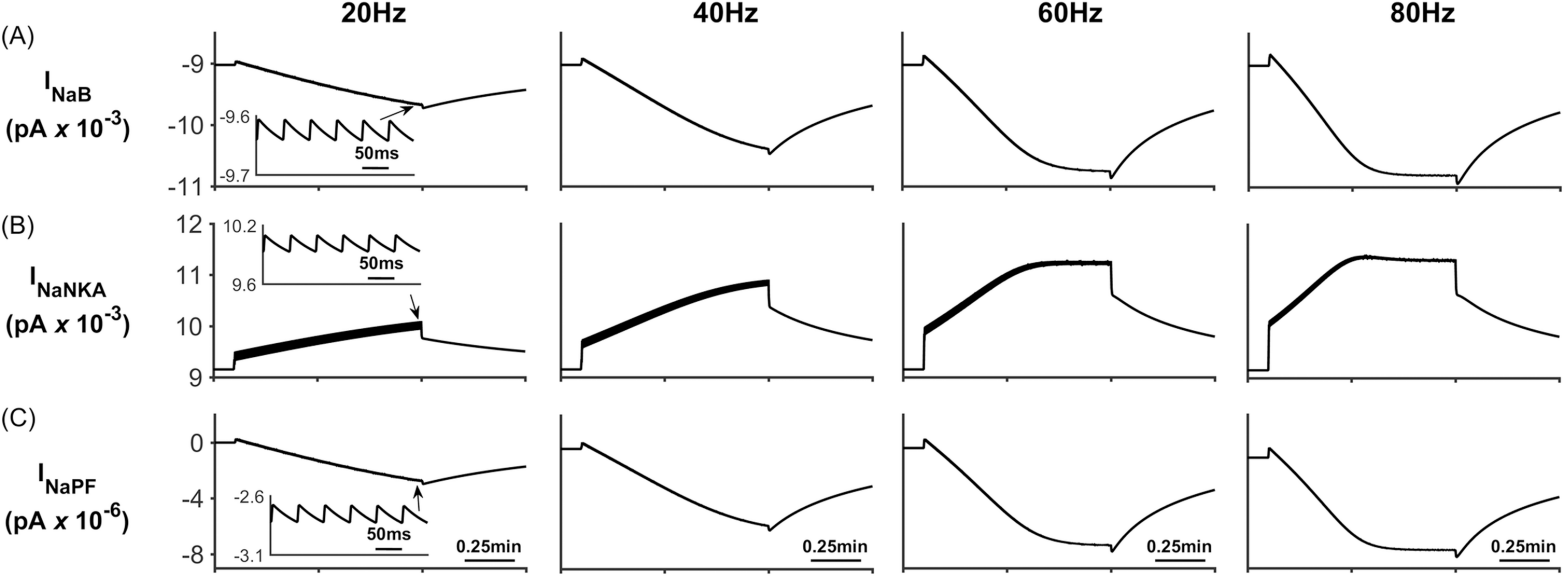
Perisynaptic Na^+^ currents. (A) Background Na^+^ current. (B) NKA current. (C) Na^+^ current along the process. During neural activity, the NKA pumps Na^+^ from the cell to allow for K^+^ uptake, therefore there is a net decrease in [Na^+^]_PsC_. When the neurone stops firing, NKA slows down and there is a net uptake of Na^+^ via the remaining channels.

Fig 7 shows the Na^+^ currents for the four different stimulus frequencies. All Na^+^ channels, except the NKA (Fig 7B) Na^+^ current, result in Na^+^ influx to the PsC. When the neurone stops firing there is a net influx of Na^+^ into the PsC. The decrease in I_NaB_ (Fig 7A) can be explained as follows: Since I_NaB_ is dependent on the astrocyte membrane potential as well as Na^+^ gradient there is a sharp decrease in the current due to the astrocyte membrane potential depolarising.

### Glutamate driven PsC Na^+^ microdomain formation

**Fig 8:**
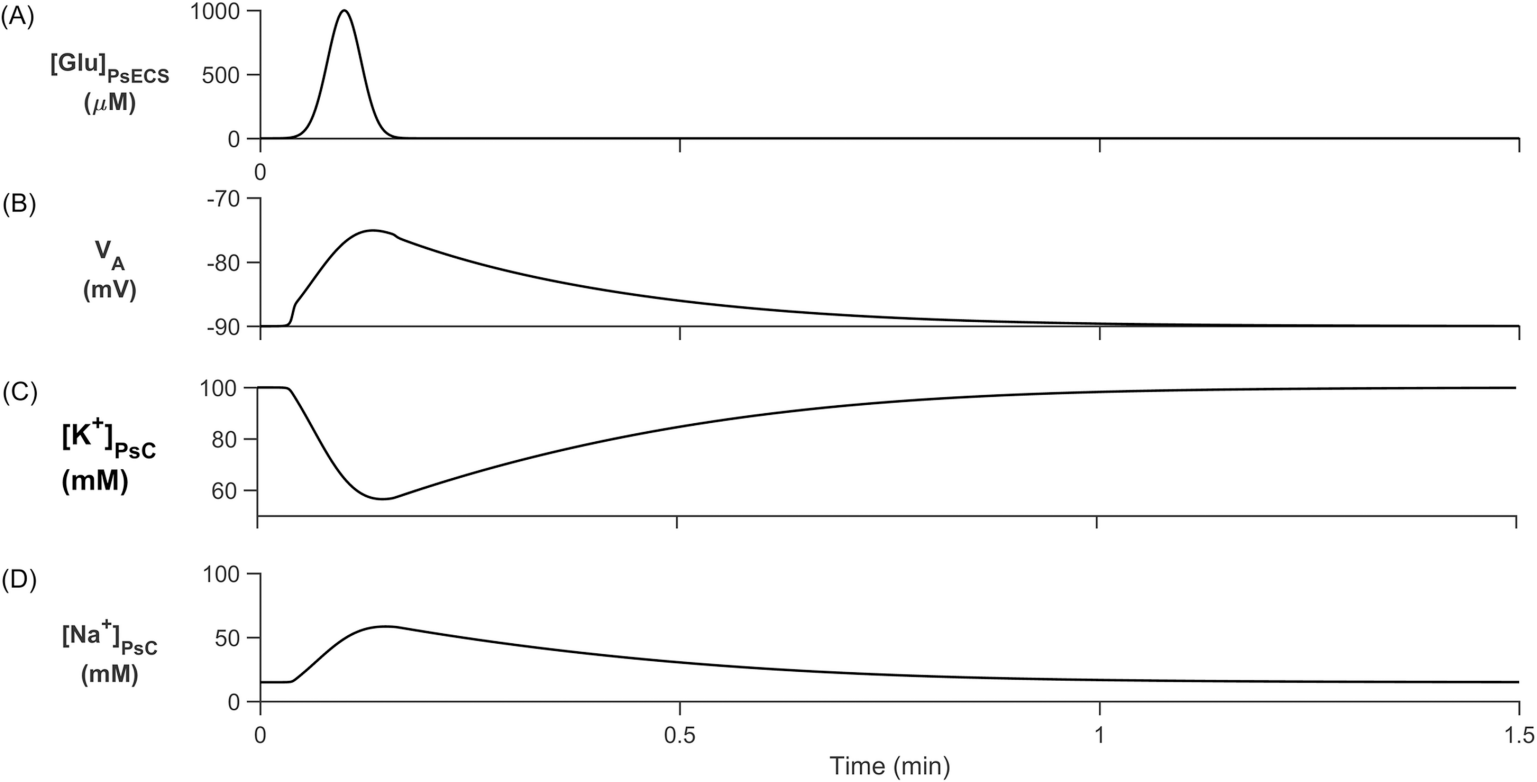
Cell membrane voltage and concentrations. (A) Glutamate is injected into the PsECS with a Gaussian distribution for ~10s with a maximum concentration of 1000μM. (B) PsC membrane voltage depolarises with ionic changes in the PsC. (C) [Glu]_PsECS_ increase causes EAAT1/2 activation and a thereby removing K^+^ and (D) the uptake of Na^+^.

As well as K^+^ buffering, astrocytes also provide a critical role in glutamate uptake and recycling via the glutamate-glutamine cycle (GGC) [57]. In this simulation, the role of glutamate transport via EAAT1/2 is investigated and results show that the slow leakage of Na^+^ ions in the astrocyte process causes Na^+^ to increase in the PsC before being returned to the PsECS via the NKA. These results support previously published experimental work [23]: there is no neuronal excitation and therefore the concentration of K^+^ in the PsECS is held constant. The concentration of glutamate in the PsECS was modulated using a Gaussian function as shown in Fig 8A. Fig 8B–8D presents the results of the PsC ionic [K^+^]_PsC_ and [Na^+^]_PsC_ concentrations and membrane voltage, V_A_, for this simulation.

**Fig 9:**
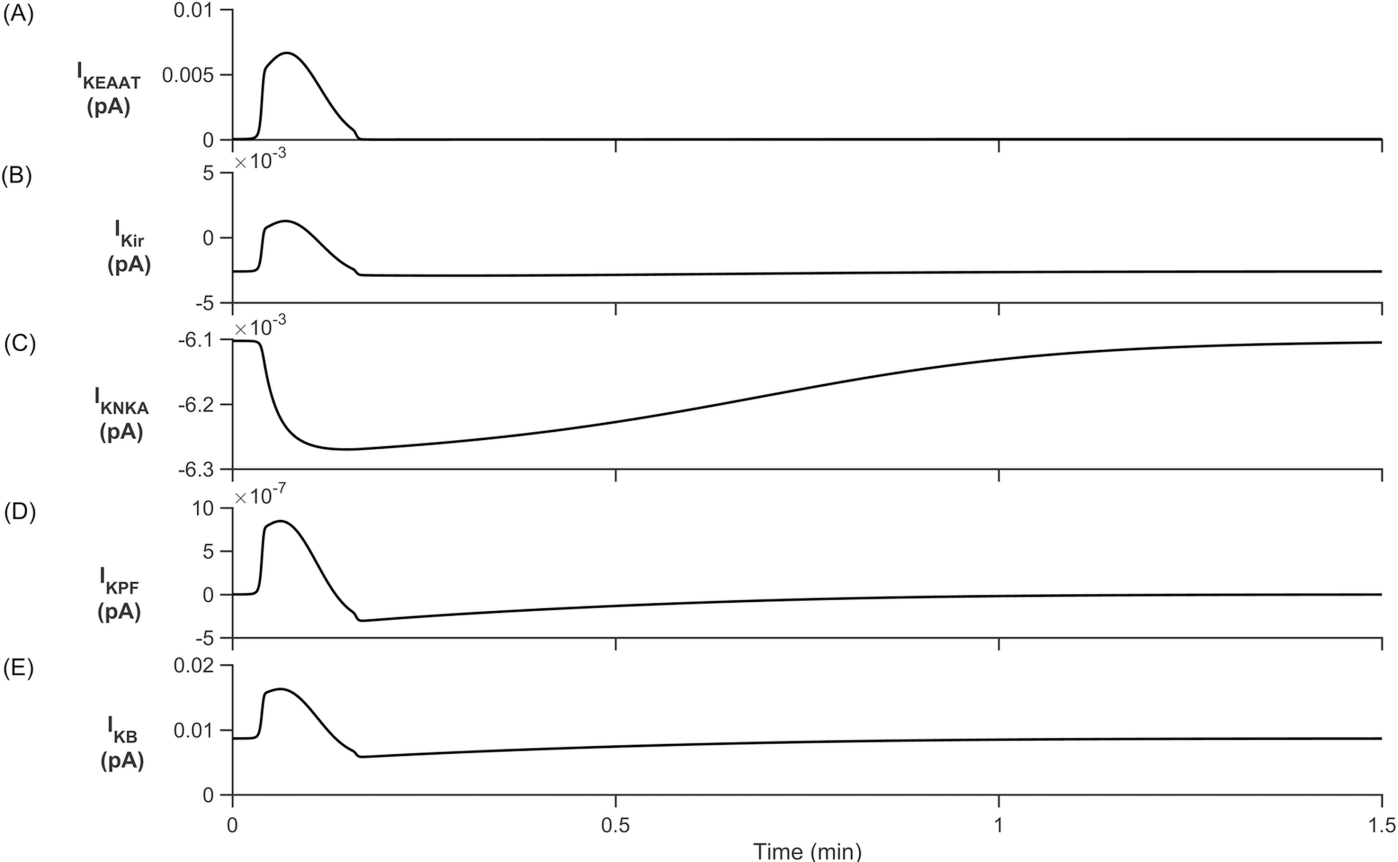
Perisynaptic K^+^ currents. (A) K^+^ EAAT1/2 current. (B) K^+^ K_ir_ current. (C) K^+^ NKA current. (D) K^+^ current along the process. (E) K^+^ background current.

**Fig 10:**
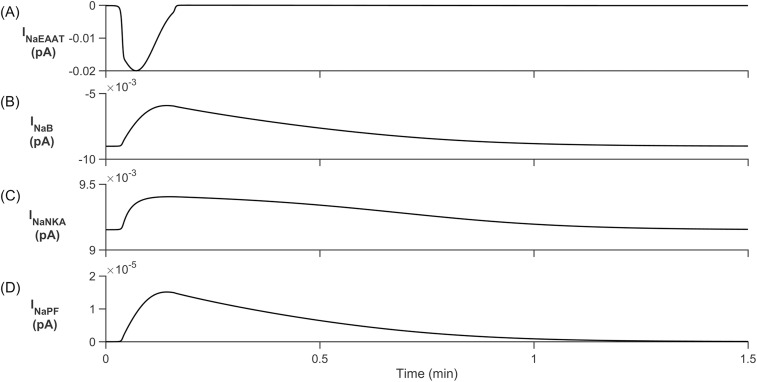
Perisynaptic Na^+^ currents. (A) Na^+^ EAAT1/2 current. (B) Background Na^+^ current. (C) Na^+^ NKA current. (D) Na^+^ current in the astrocyte process. With the increase of [Glu]_PsECS_, EAAT1/2 transport rate is increased to remove glutamate from the PsECS, in turn Na^+^ is taken up. Furthermore, as [Na^+^]_PsC_ increases, the rate of Na^+^ influx from the background channel decreases. All other channels remove Na^+^ until [Na^+^]_PsECS_ reaches steady state conditions once again.

From Fig 8C and 8D we clearly see that the [K^+^]_PsC_ decreases while [Na^+^]_PsC_ increases, this is the opposite dynamics to that observed in Fig 4C and 4D. This is because K^+^ in the PsECS is now held constant at 3 mM and therefore all K^+^ channels except the NKA and slow leakage through the astrocyte process remove K^+^ from the PsC (Fig 9) resulting in a net K^+^ efflux. The main driving force behind Na^+^ uptake by the PsC is the EAAT1/2 transporter which is also responsible for the removal of glutamate from the PsECS (Fig 10).

During [Glu]_PsECS_ injection, the EAAT1/2 and K_ir_ release K^+^ at an accelerated rate. This is opposed by NKA and the transport of K^+^ from the astrocyte soma to the PsC. When glutamate falls to baseline levels, the EAAT1/2 and K_ir_ channels quickly revert to their initial rates. NKA and transport of K^+^ from the astrocyte soma is then able to establish baseline ionic concentrations at the PsC.

As in the previous simulation, retention of Na^+^ ions as they flow within the astrocyte process substantially limits the transport rate of these ions away from the PsC. In this case, Na^+^ is restricted and therefore a Na^+^ microdomain forms at the PsC. Note: similar to the results presented in [23] there is a long decay (~80s) transient of Na^+^ which far outlasts the glutamate signal decrease (Fig 8D) and we propose that this is due to the slow removal of Na^+^ by the NKA. These observations could explain previously observed experimental results [23].

### ECS K^+^ and Glu driven PsC microdomain formation

**Fig 11:**
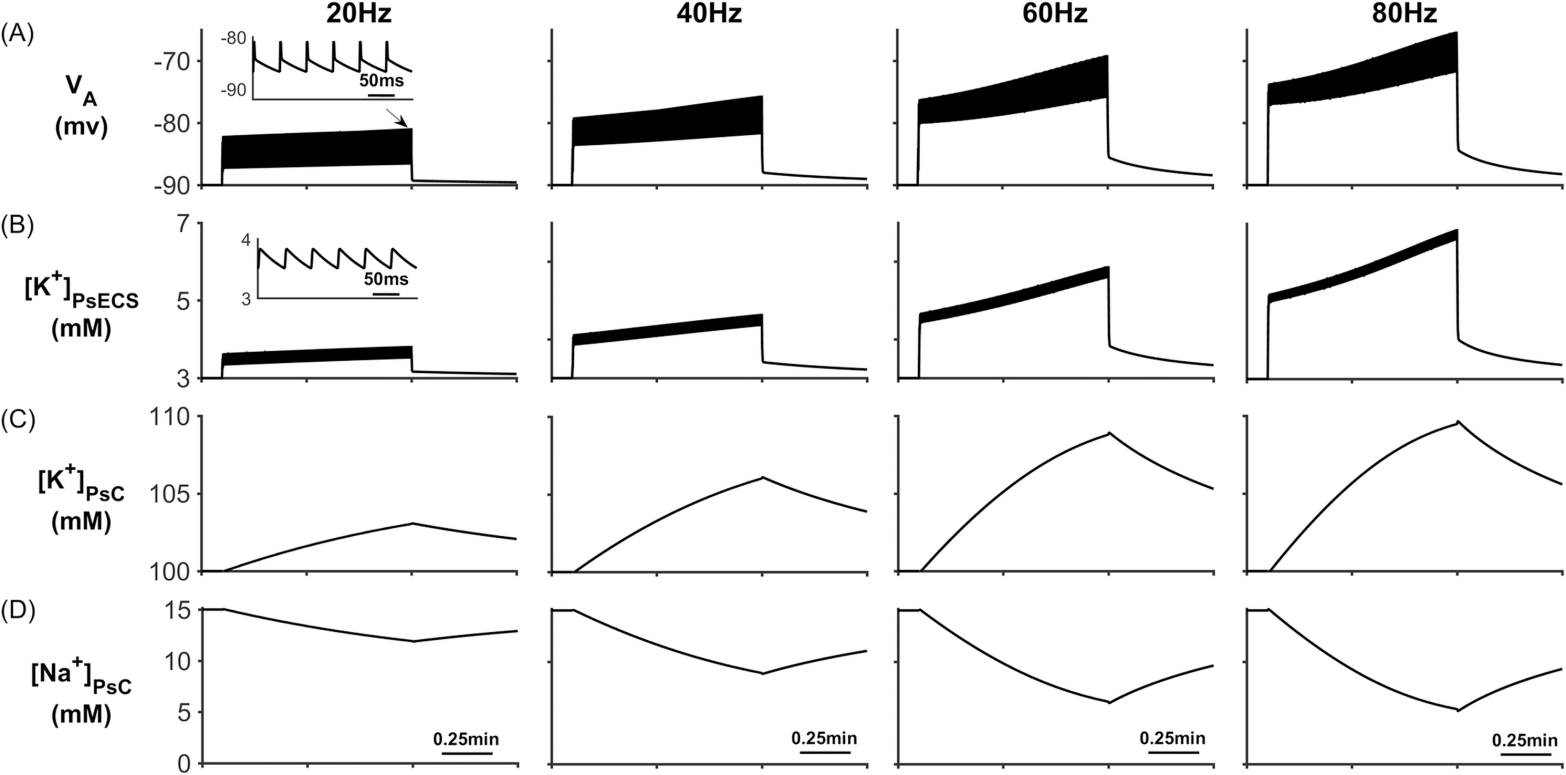
PsC membrane voltages and concentrations against time. (A) Astrocyte membrane voltage (V_A_). (B) [K^+^]_PsECS_. (C) [K^+^]_PsC._ transient. (D) [Na^+^]_PsC_ transient.

**Fig 12:**
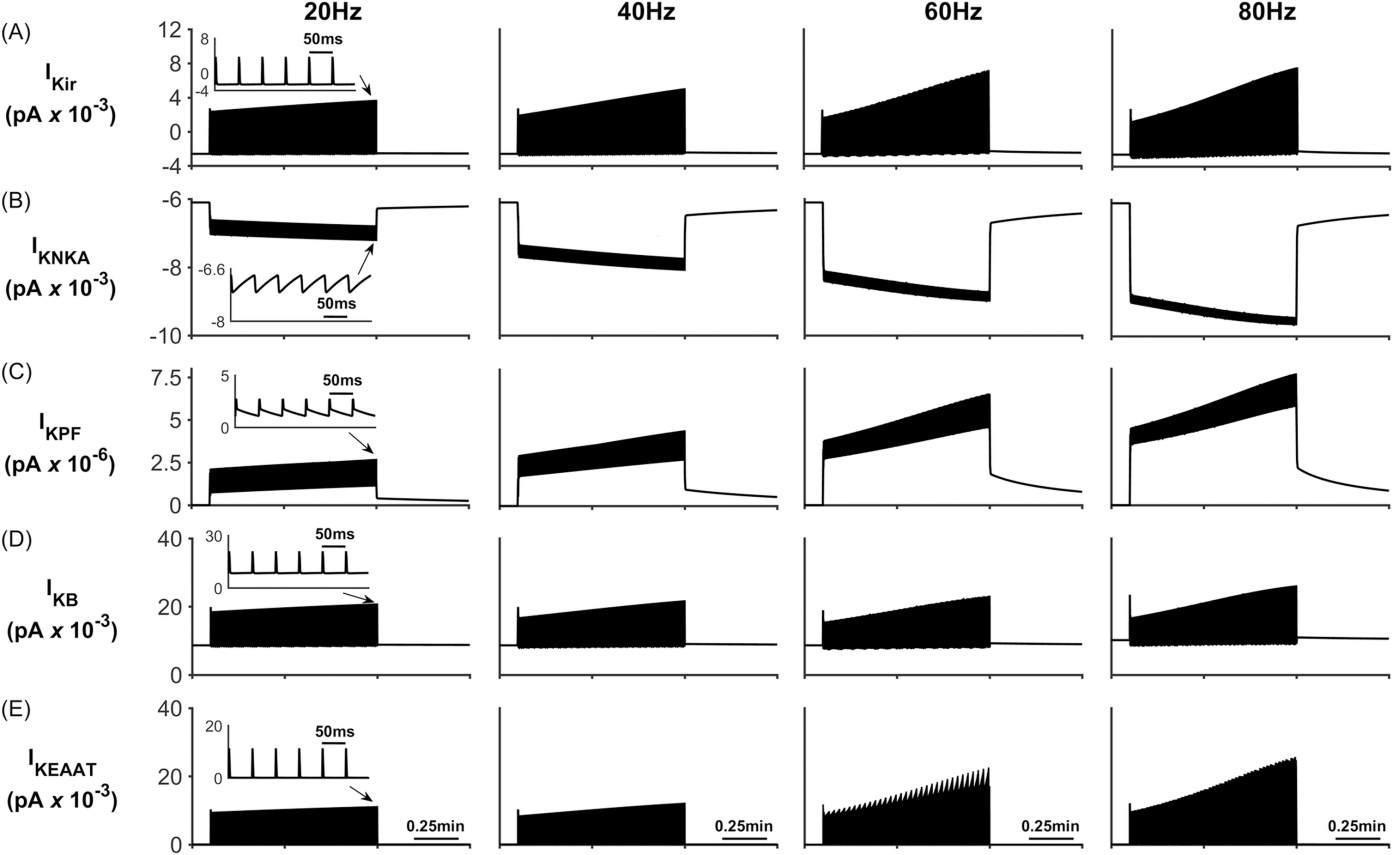
Perisynaptic K^+^ currents. (A) K^+^ K_ir_ current. (B) K^+^ NKA current. (C) K^+^ current along the process. (D) Background K^+^ current. (E) K^+^ EAAT current. Note: similar to the first simulation, as the neurone firing rate increases the magnitude of all currents also increase.

**Fig 13:**
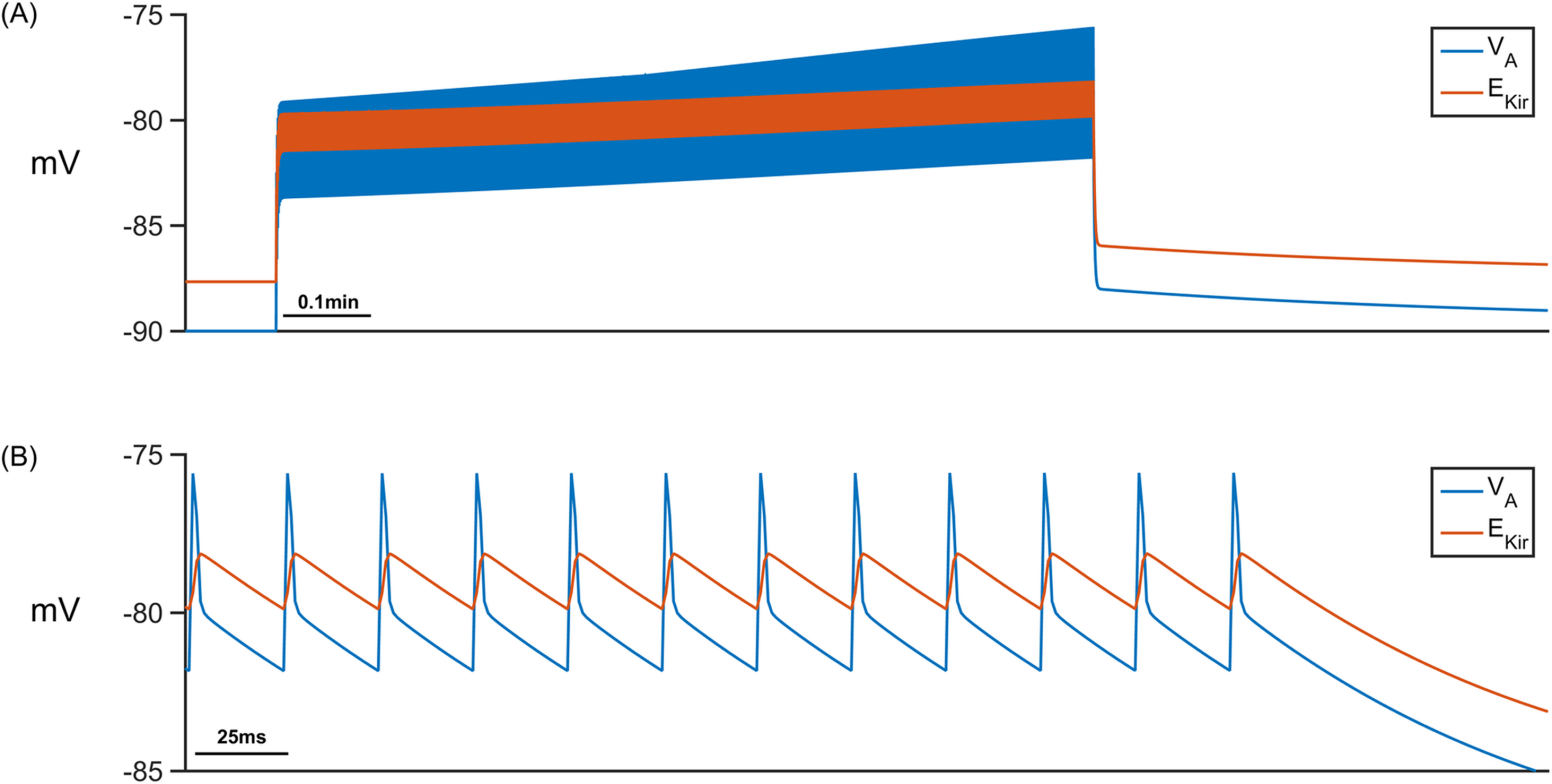
K_ir_ reversal (neurone firing rate: 40Hz). (A) The astrocyte membrane voltage (blue) and the K_ir_ reversal potential continually cross over during neurone stimulation. This results in periodic reversal of the K_ir_ channel. (B) Magnification of A for the last few hundred milliseconds of stimulation.

The previous two simulations have shown that K^+^ or Na^+^ microdomains form in the PsC when the system is stimulated with PsECS changes in K^+^ or Glu respectively. However, while these simulations show that our hypothesis could potentially explain experimental observations, we now wish to use our model to predict ionic dynamics at the PsC under physiological conditions where both K^+^ and Glu are released at the presynaptic terminal. In this case K^+^ is released by the neurone as before and a 100 μM puff of Glu is released into the PsECS, with each spike event. Presynaptic neurone firing rates are 20Hz, 40Hz, 60Hz and 80Hz, for a period of 0.1min to 1min. The results presented in Fig 11 show that the overall behaviour of the model, i.e. microdomain formation of K^+^ in the PsC, occurs. However, the astrocyte membrane voltage V_A_ oscillates (~7mV amplitude) (Fig 11A) caused by the periodic reversal of the K_ir_ channel (See Fig 12A). This reversal is caused by the efflux of K^+^ via the EAAT1/2 (Fig 12E) channel. Moreover, the dynamic behaviour of the reversal potential of the K_ir_ and V_A_ continuously cause reversal of the overall polarity (Fig 13), thus causing the K_ir_ channel to periodically reverse direction resulting in an efflux of K^+^ into the ECS; this can be seen as oscillations in [K^+^]_PsECS_. It can be observed in Fig 11C and 11D that a K^+^ microdomain is formed in the PsC and its magnitude increases with frequency while the magnitude of Na^+^ reduces. This is due to the behaviour of the K^+^ uptake by NKA dominating over the K^+^ efflux pathways (See Fig 12).

**Fig 14:**
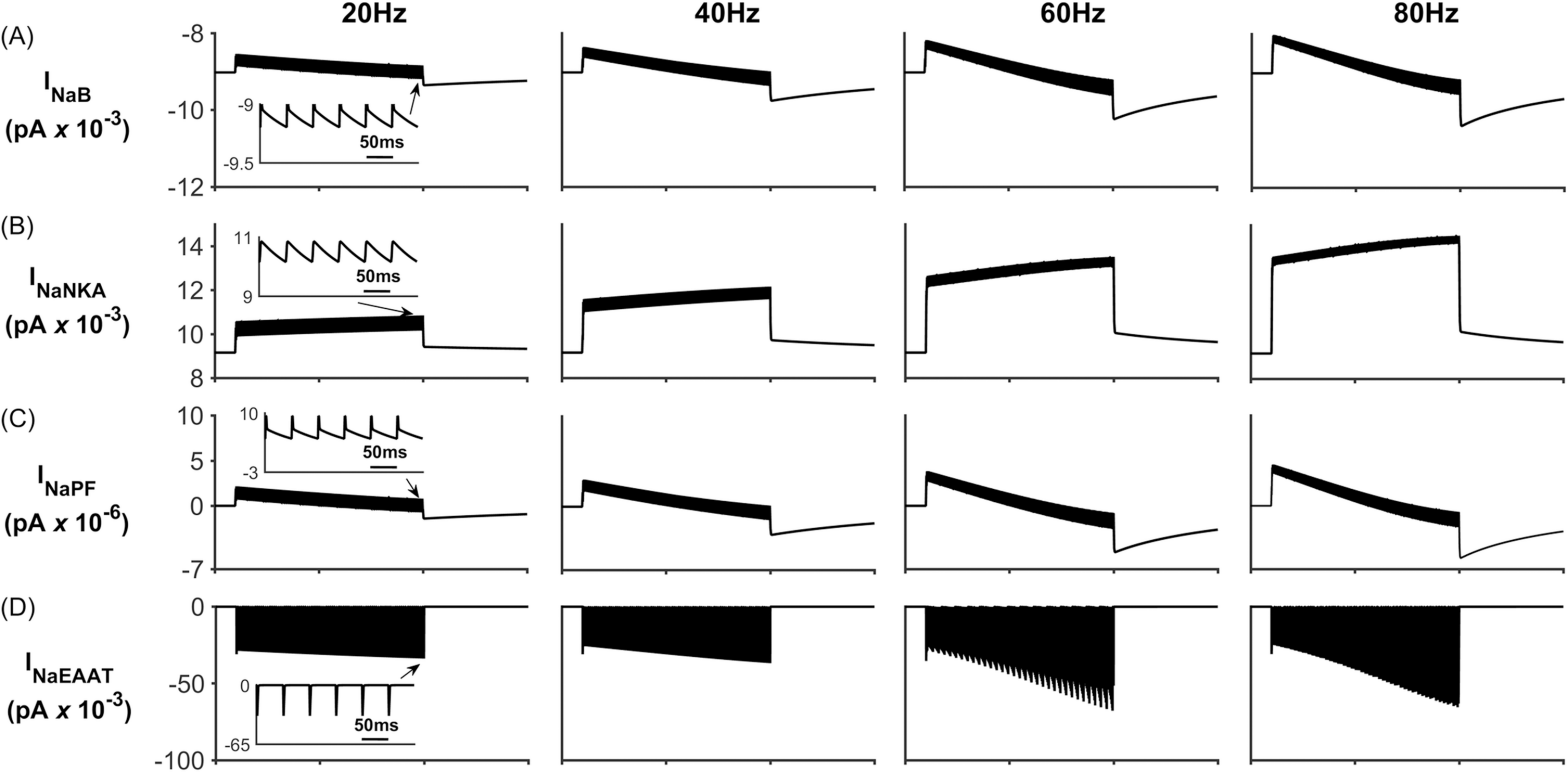
Perisynaptic Na^+^ currents. (A) Background Na^+^ current. (B) NKA current. (C) Na^+^ current along the process. (D) EAAT Na^+^ current During neural activity, the NKA pumps Na^+^ from the cell to allow for K^+^ uptake, therefore there is a net decrease in [Na^+^]_PsC_. When the neurone stops firing, NKA slows down and there is a net uptake of Na^+^ via the remaining channels.

Fig 14 shows the Na^+^ currents for the four different stimulus frequencies. As expected all Na^+^ channels on the PsC membrane, except the NKA (Fig 14B) result in Na^+^ influx to the PsC. I_NaEAAT_ has a large peak amplitude for a short duration (few milliseconds) due to the EAAT channel slowing down after removal of Glu from PsECS.

### Parameter sensitivity

Having analysed the formation of microdomains and model behaviour in the previous three simulations we now explore the sensitivity of the model to model parameters. These parameters are PsC surface area, the maximum NKA pump rate, P_max_, and the potential barrier to ion flow along the process, φ_w_. In these simulations a neuronal firing rate of 40Hz was chosen.

**Fig 15:**
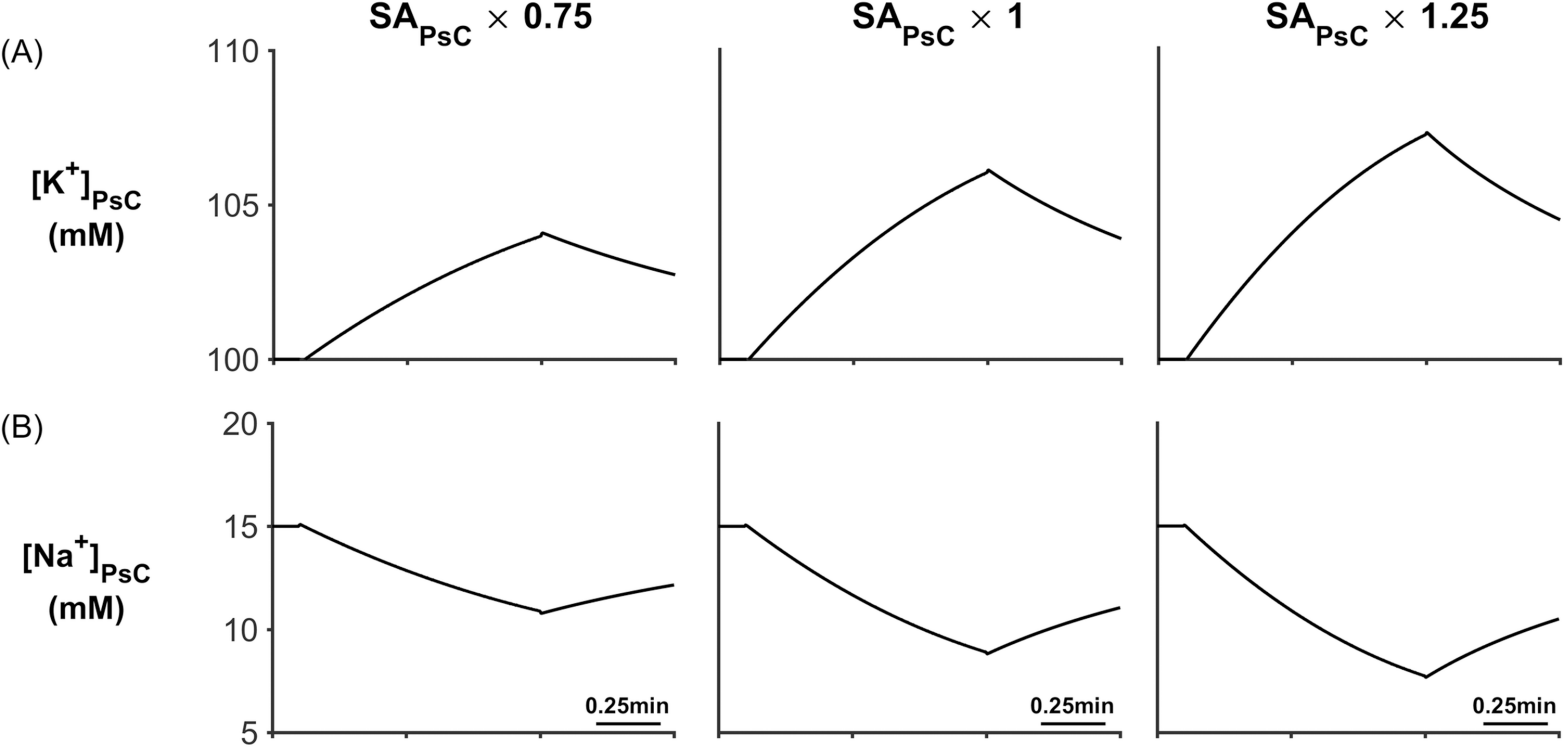
Microdomain formation for different PsC surface areas. (A) PsC K^+^ concentration. (B) PsC Na^+^ concentration. As the PsC surface area increases so the amplitude of the K^+^ microdomain increases and the Na^+^ microdomain amplitude decreases.

Microdomain Sensitivity to PsC Surface Area (SA). Three different values of PsC SA were chosen for this simulation; PsC SA × 0.75, PsC SA × 1 and PsC SA × 1.25. The results of these simulations are shown in Fig 15 where it can clearly be seen that the amplitude of the K^+^ microdomain increased with PsC SA with a corresponding drop in the concentration of Na^+^. Also, the K^+^ and Na^+^ currents efflux/influx also increased with PsC SA (See Supplementary S1 Fig for the changes in K^+^ currents).

**Fig 16:**
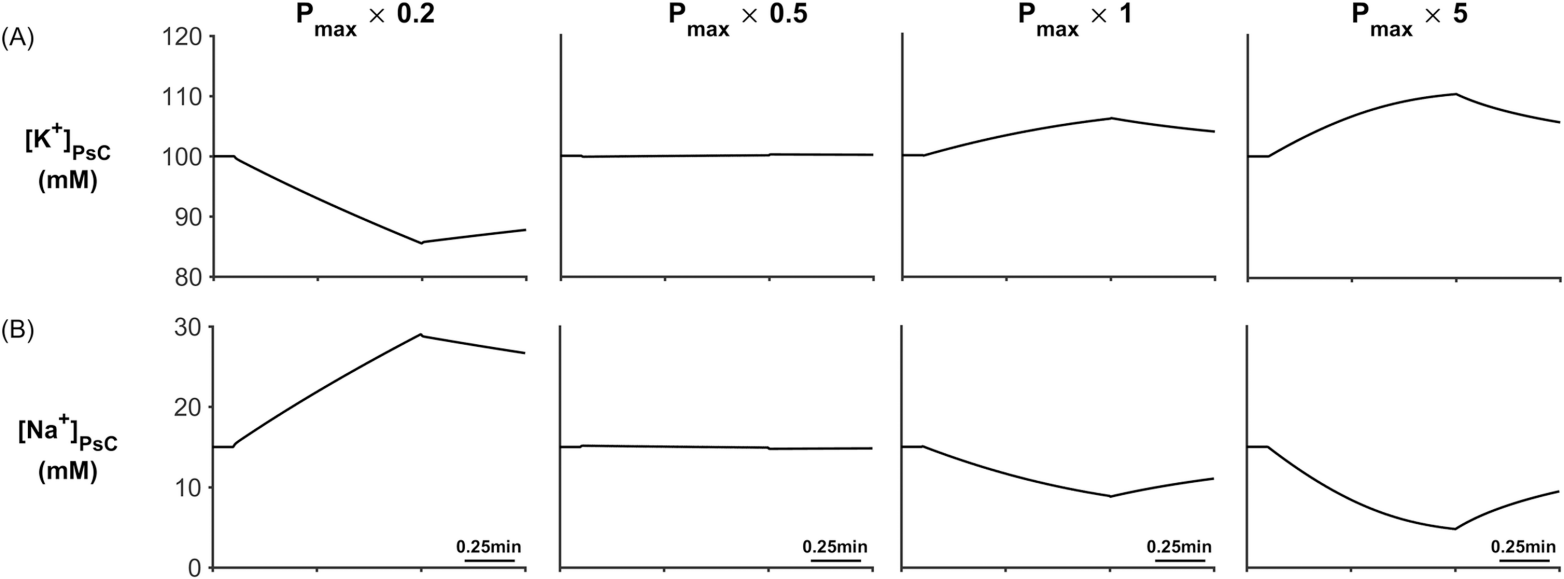
Microdomain formation for different values of NKA maximum pump rate: (A) [K^+^]_PsC_ as a function of Pmax and (B) [Na^+^]_PsC_ as a function of Pmax.

Microdomain Sensitivity to P_max._ Four different values of P_max_ were chosen for this simulation; P_max_ × 0.2, P_max_ × 0.5, P_max_ × 1 and P_max_ × 5. The results of these simulations are shown in Fig 16 where it can clearly be seen that [K^+^]_PsC_ and [Na^+^]_PsC_ is strongly dependent on P_max_. Using the P_max_ x 0.2 value causes [K^+^]_PsC_ to decrease and [Na^+^]_PsC_ to increases and as P_max_ increases, [K^+^]_PsC_ begins to form a microdomain with [Na^+^]_PsC_ steadily decreasing. From these simulations we can conclude that when the NKA pump rate is low it is no longer the dominant co-transporter and both the EAAT co-transporter and K_ir_ channel dictate [K^+^]_PsC_ and [Na^+^]_PsC_ dynamics. The opposite is true when the pump rate is large.

**Fig 17:**
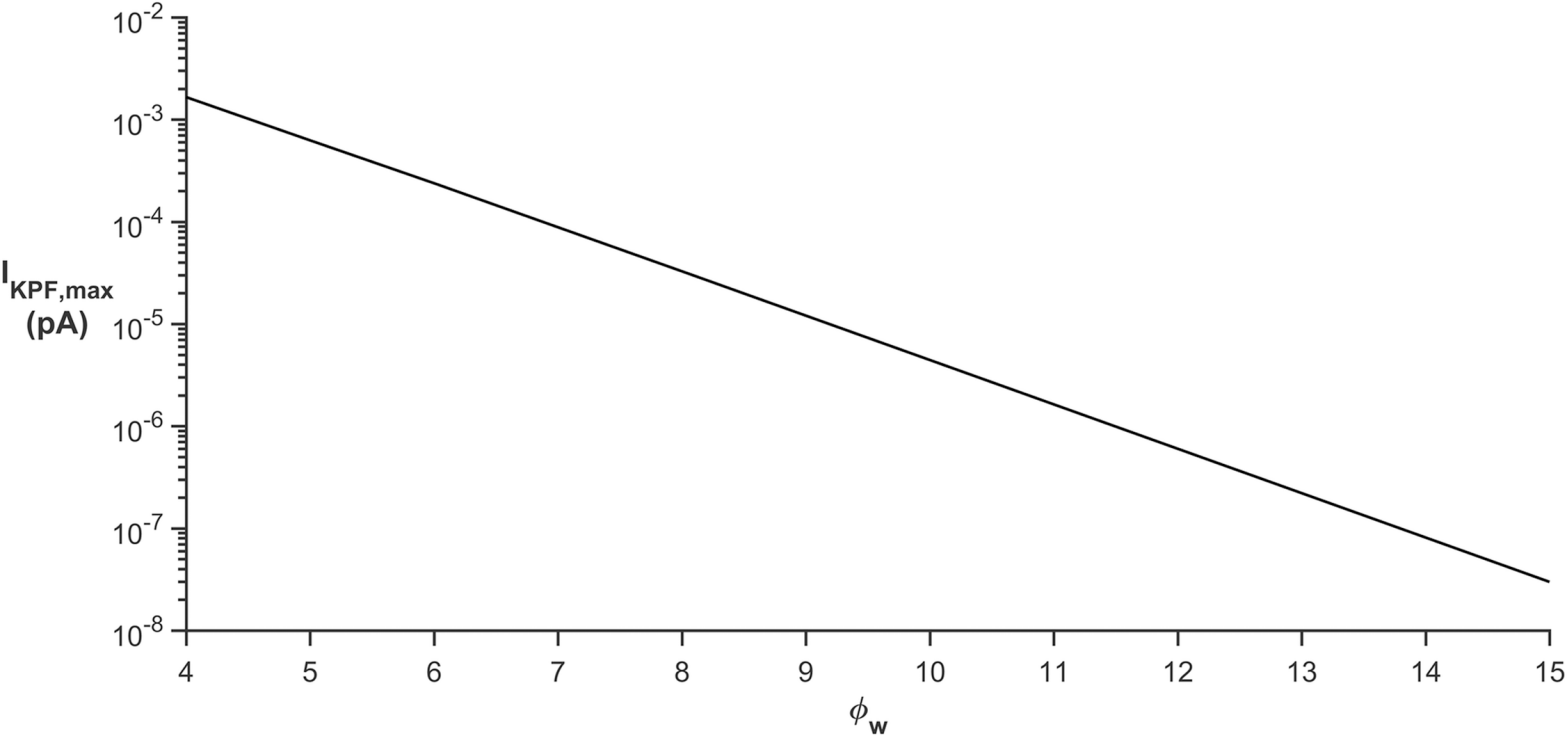
Peak K+ current along the process for different values of φ_w_.

Microdomain Sensitivity to φ_w._ In this simulation φ_w_ was varied from 4 k_B_T to 15 k_B_T. Fig 17 shows the peak K^+^ current along the process for the different values of φ_w_. As φ_w_ is decreased, the peak current along the process increases exponentially. Therefore, with decreasing φ_w_ the formation of a microdomain becomes less likely as I_KPF,max_ is increasing and eventually I_KPF,max_ approaches an electro-diffusion limited model with no likelihood of a microdomain forming at the PsC.

From these simulations it is clear that the mechanism responsible for the formation of microdomains is the well formation along the process which effectively semi-isolate the PsC from the astrocyte soma when φ_w_ is 10 k_B_T or greater. It is also clear that the PsC SA can limit the maximum amplitude of the microdomain concentration. This is due to the increase/decrease of ion channel densities on the membrane of the PsC. Moreover, the NKA maximum pump rate also has an important role in the formation of microdomains whereby if the pump rate is low then K^+^ clearance by NKA weakens; effectively these to ion transporters compete to move K^+^ and Na^+^ ion across the membrane but in opposite directions.

## Discussion

Homeostatic control over synaptic cleft, of which K^+^ buffering and glutamate uptake is of the most fundamental importance, represent the quintessential function of astroglial cradle formed by the perisynaptic process [7, 8]. Physiological K^+^ buffering is essentially K^+^ recycling between astroglial and neuronal compartments: NKA-dependent astroglial uptake limits the peak of extracellular K^+^ rise, whereas K^+^ efflux is imperative for restoration of [K^+^]_i_ in neuronal terminal [3, 10]. Glutamate uptake, as well as glutamate conversion into glutamine and glutamine shuttling to neuronal terminals are regulated by Na^+^ concentration in the cytosol of astroglia; astroglial [Na^+^]_i_ in addition controls a multitude of SLC (Solute Carrier) transporters responsible for various homeostatic pathways [20, 21]. Both mechanisms require localisation of ionic signalling within the confines of astroglial synaptic cradle, and indeed local and long-lasting [Na^+^]_i_ transients are routinely recorded from astroglial processes [23, 58]. Molecular machinery responsible for localisation of [K^+^]_i_ and [Na^+^]_i_ increases remains unknown.

Here we table a novel mechanism of localisation of ionic signals in astroglial cells. We propose that ion retention within thin astrocyte processes can give rise to the formation of K^+^ and Na^+^ microdomains at the PsC. This localisation of astroglial ionic microdomains arises because in thin processes, surface conduction dominates over volume conduction, and because membrane lipids are negatively charged, deep potential wells form near the dipole heads restricting the flow of cations along the process. Therefore, cations must hop from well to well which restricts ion conduction along the membrane. This hopping effectively semi-isolates the PsC from the astrocytic main body allowing the formation of K^+^ and Na^+^ microdomains at the PsC under different conditions.

We modelled ionic responses of the PsC to the neuronal excitation that results in an increase in K^+^ concentration in the synaptic cleft. For the simulation, glutamate in the cleft was held constant at the background level and, during neuronal excitation, K^+^ was released into the PsECS leading to a depolarisation in the PsC membrane voltage due to ionic currents flowing through the PsC membrane. The simulations demonstrate that a K^+^ microdomain formed at the PsC due to the restricted flow of these ions along the astrocyte process. We further contemplate that the K^+^ microdomain provides the driving force for the return of K^+^ to the PsECS via background K^+^ channels for uptake by the neurone via its NKA. Essentially, K^+^ is transiently “stored” at the PsC during neuronal excitation where it decreases the electrochemical gradient of K^+^, so reducing inward flow of potassium through K_ir_; this “stored Potassium” is then available to replenish neuronal K^+^ levels when the excitation ceases, thereby preventing K^+^ undershoot in the extracellular space. These observations are consistent with in vivo experimental data [59], and partly explain why inward rectifying K^+^ channels may play a prominent role for K^+^ uptake at large volume glial processes (e.g. terminal endfeet of retinal Muller cells [60] but not at low volume perisynaptic cradles. These results will also necessitate a reappraisal of the mechanisms and role of astrocytes in potassium accumulation during seizure activity [61], especially given the observation that loss of function mutations in the gene encoding K_ir_4.1 are associated with a human epilepsy syndrome [62] and astrocyte K_ir_4.1 expression is decreased in acquired epilepsy models [63]. Moreover, other conditions that have been proposed to be due to abnormalities of potassium homeostasis such as familial hemiplegic migraine are associated with mutations of the gene encoding the alpha2 subunit of NKA, which is predominantly expressed in astrocytes [64].

Our model also shows that the influx of Na^+^ ions into the astrocyte process causes a Na^+^ microdomain to form at the PsC where the decay rate of Na^+^ is governed by the NKA. In this simulation, there is no neuronal excitation and the concentration of glutamate in the cleft was modulated using a Gaussian function and was taken up at the PsC by EAAT1/2. A slow decay of Na^+^ was observed after the glutamate uptake ceased which is in strong agreement with experimental observations [21, 23].

In summary, we accept our model for ion retention in thin astrocyte processes requires much more refinement. For example, the main challenge would be to account for the dynamic interaction between the charge present in membrane proteins and the charged ions in the astrocyte medium. Additionally, the dimensions of the astrocyte are such that the membrane proteins are unlikely to be represented by point charges and a more atomistic view of the proteins would need to be found to create a map of the charge distribution at the atomic scale. Also, any simulations would require a large number of atoms to be taken into account to obtain the electrostatic potential profile at the membrane-cytoplasm interface. Once the electrostatic potential in thin process is found and combined with the cation distribution, then the movement of ions can be modelled. Moreover, we have only considered K^+^ and Na^+^ ions in our model and therefore a more biophysical model would need to consider Ca^2+^ microdomains and Cl^-^ ion dynamics with the inclusion of the associated membrane transporters such as Sodium/Calcium exchanger and Sodium/Potassium/Chloride cotransporter.

We have assumed throughout the model an infinite GECS but this in reality would not be the case. However more biological data about the shape and size of extracellular spaces and morphology of the perisynaptic cradle would be required before modelling in such a way. Despite this our model does however indicate that the morphological and biophysical properties of the astroglial perisynaptic processes facilitate emergence of Na^+^ and K^+^ microdomains that are essential for astroglial homeostatic support of synaptic transmission in the central nervous system, and point to new and important implications for potassium homeostasis during pathological activity such as seizures. For example, the long-held view that spatial potassium buffering plays an important role in seizure activity has been challenged by a number of experimental observations including only a small effect on potassium buffering of knocking out astrocytic gap junction proteins [65], and an antiepileptic effect of gap junction blockers [66]. Indeed, it has been proposed that gap junctions are not necessary for potassium buffering but instead are important for maintaining neuronal metabolism during seizure activity [67]. Our model supports the experimental data and indicates that potassium buffering is a locally restricted phenomenon, and therefore our model challenges the orthodox view of the role of glial spatial potassium buffering during pathological activity. Indeed, our model supports the existence of a mechanism that prevents local potassium depletion during excessive neuronal firing and indicates a novel mechanism by which astrocytes maintain neuronal excitability during pathological activity.

Finally, we would like to point out that ion hopping is a current transport mechanism that involves ions surmounting potential barriers. It is therefore strongly temperature dependent, following Arrhenius behaviour, and has a distinctive temperature and voltage dependence or 'signature' that enables identification against other possible current mechanisms; this may provide a means to test our hypothesis as the kinetics of ionic microdomains formed by Na^+^, K^+^ or even Cl^-^ in perisynaptic processes could be quantified in experimental brain slices using respective intracellular probes.

## Supporting information

S1 Text Neurone model. Description of the Hodgkin and Huxley neurone model used in the model simulations.
(DOCX)

S1 Table Neurone parameters. List of parameter values used by the Neuron Model.
(DOCX)

S1 Fig Sensitivity to PsC surface area, perisynaptic K^+^ currents. (A) K^+^ K_ir_ current. (B) K^+^ NKA current. (C) K^+^ current along the process. (D) Background K^+^ current. (E) K^+^ EAAT current.
(DOCX)
