# Supplementary material for: Potassium and sodium microdomains in thin astroglial processes: A computational model study
Source: PLoS Comput Biol. 2018 May 18;14(5):e1006151. doi: 10.1371/journal.pcbi.1006151 (PMC5979043; doi:10.1371/journal.pcbi.1006151)
Supplement: S1 Text — Description of the Hodgkin and Huxley neurone model used in the model simulations. (DOCX) [file pcbi.1006151.s001.docx]

# S1 Text: Neurone Model

The core of the neuronal model utilised in the work consists of the biophysical Hodgkin and Huxley (HH) type model. All parameter values for the model can be found in supplementary material S2 Table.

## Membrane voltage

The membrane potential of the neuron is described by:

$C_{m}\frac{dV_{\mathrm{Neu}}}{\mathrm{dt}}= {- g}_{\mathrm{NaNeu}}m^{3}h\left( V_{\mathrm{Neu}}-E_{\mathrm{NaNeu}} \right){- g}_{\mathrm{KNeu}}n^{4}\left( V_{\mathrm{Neu}}-E_{\mathrm{KNeu}} \right){- g}_{\mathrm{LNeu}}\left( V_{\mathrm{Neu}}-E_{\mathrm{LNeu}} \right)$ (1)

Where C_m_ is the membrane capacitance, g_NaNeu_ is the Na^+^ channel conductance, g_KNeu_ is the K^+^ channel conductance, g_LNeu_ is the leak channel conductance, V_Neu_ is the neuron membrane voltage with an initial condition of -0.01 volts, E_NaNeu_, E_KNeu_ and E_LNeu_ are the Na^+^ channel, K^+^ channel and Leak channel reversal potential respectively and m, n and h are channel gating variables.

The Na^+^ activation variable is given by:

$\frac{\mathrm{dm}}{\mathrm{dt}}= \alpha_{m}\left( 1-m \right){- \beta}_{m}m$ (2)

where

$\alpha_{m}= 0.1\frac{V_{\mathrm{Neu}}+40}{1-exp\left( -\left( \frac{V_{\mathrm{Neu}}+40}{10} \right) \right)}$ (3)

and

$\beta_{m}=4exp\left( -\left( \frac{V_{\mathrm{Neu}}+65}{18} \right) \right)$ (4)

The Na^+^ inactivation variable is given by:

$\frac{\mathrm{dh}}{\mathrm{dt}}= \alpha_{\mathrm{mh}}\left( 1-h \right){- \beta}_{h}h$ (5)

where

$\alpha_{h}= 0.07exp\left( -\left( \frac{V_{\mathrm{Neu}}+65}{20} \right) \right)$ (6)

and

$\beta_{h}=0.1\frac{1}{\exp\left( -\left( \frac{V_{\mathrm{Neu}}+35}{10} \right) \right)+1}$ (7)

The K^+^ activation variable is given by

$\frac{\mathrm{dn}}{\mathrm{dt}}= \alpha_{n}\left( 1-n \right){- \beta}_{n}n$ (8)

where

$\alpha_{n}= 0.01\frac{V_{\mathrm{Neu}}+55}{1-exp\left( -\left( \frac{V_{\mathrm{Neu}}+55}{10} \right) \right)}$ (9)

and

$\beta_{n}=0.125exp\left( -\left( \frac{V_{\mathrm{Neu}}+65}{80} \right) \right)$ (10)

## Neuron Potassium Channel (K_Neu_)

The HH model simulates current flow of K^+^ through a voltage gated channel, therefore the current flow of K^+^ from the neuron can be modelled as:

$I_{\mathrm{KNeu}}={- g}_{\mathrm{KNeu}}n^{4}\left( V_{\mathrm{Neu}}-E_{\mathrm{KNeu}} \right)\mathrm{SA}_{\mathrm{Syn}}$ (11)

where SA_syn_ is the surface area of the synapse.
