# Supplementary material for: Potassium and sodium microdomains in thin astroglial processes: A computational model study
Source: PLoS Comput Biol. 2018 May 18;14(5):e1006151. doi: 10.1371/journal.pcbi.1006151 (PMC5979043; doi:10.1371/journal.pcbi.1006151)
Supplement: S1 Table — List of parameter values used by the Neuron Model. (DOCX) [file pcbi.1006151.s002.docx]

# S1 Table: Neurone Parameters

| **Parameter** | **Value** | **Units** | **Description** |
| --- | --- | --- | --- |
| **g_KNeu_** | 360 | S/m^2^ | Maximum K^+^ channel conductance |
| **g_NaNeu_** | 1200 | S/m^2^ | Maximum Na^+^ channel conductance |
| **g_LNeu_** | 3 | S/m^2^ | Maximum leak channel conductance |
| **E_KNeu_** | -0.12 | V | K^+^ channel reversal potential |
| **E_NaNeu_** | 0.115 | V | Na^+^ channel reversal potential |
| **E_LNeu_** | 0.010613 | V | Leak channel reversal potential |
| **C_m_** | 0.01 | F/m^2^ | Membrane capacitance |
