# Supplementary material for: Potassium and sodium microdomains in thin astroglial processes: A computational model study
Source: PLoS Comput Biol. 2018 May 18;14(5):e1006151. doi: 10.1371/journal.pcbi.1006151 (PMC5979043; doi:10.1371/journal.pcbi.1006151)
Supplement: S1 Fig — (A) K+ Kir current. (B) K+ NKA current. (C) K+ current along the process. (D) Background K+ current. (E) K+ EAAT current. (DOCX) [file pcbi.1006151.s003.docx]

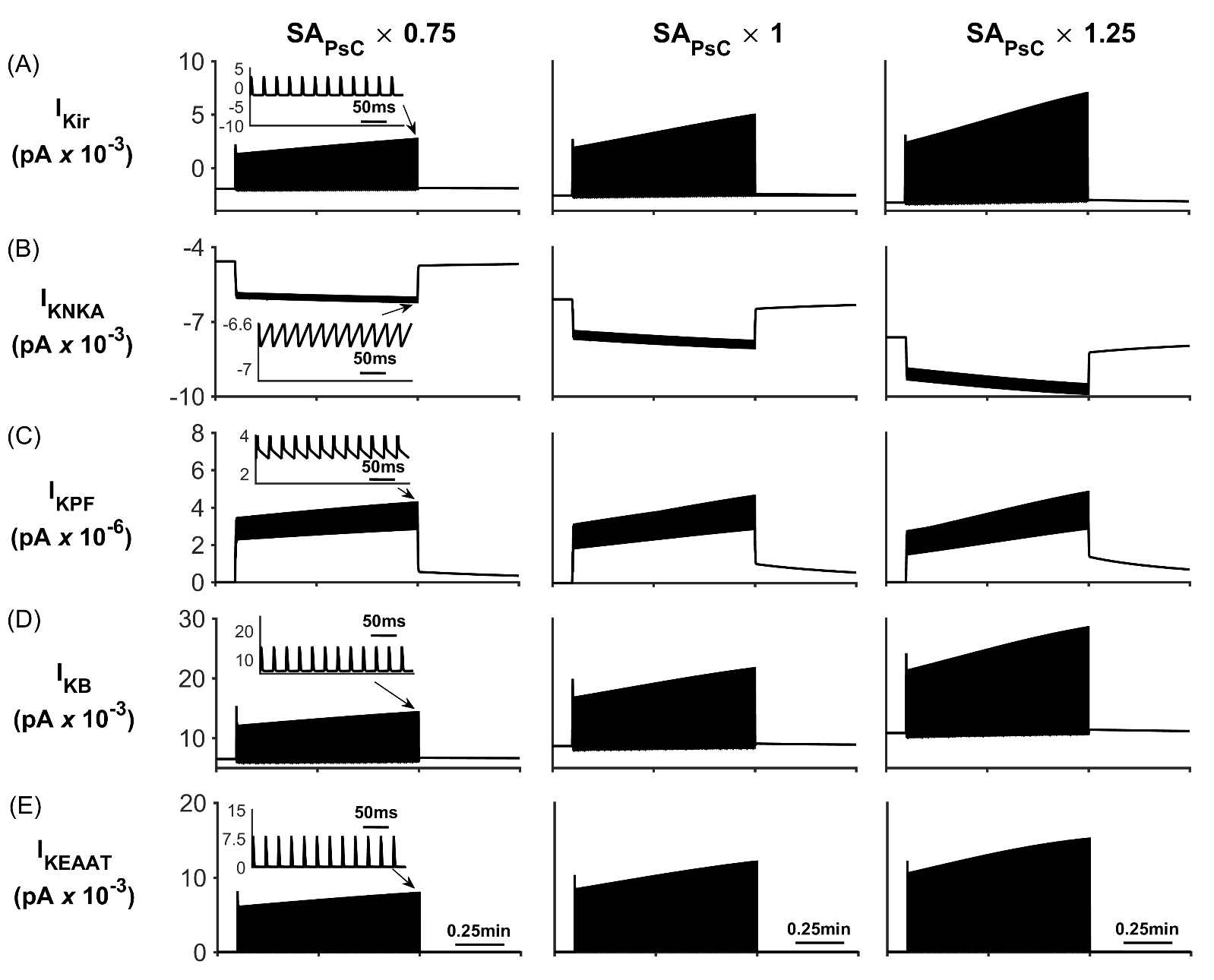


**S1 Fig. Sensitivity to PsC surface area,** **Perisynaptic K^+^ currents.** (A) K^+^ K_ir_ current. (B) K^+^ NKA current. (C) K^+^ current along the process. (D) Background K^+^ current. (E) K^+^ EAAT current.
